# Exercise timing as a potential temporal regulator of neurolymphatic physiology in neurodegenerative proteinopathies: a mechanistic framework

**DOI:** 10.3389/fnins.2026.1879617

**Published:** 2026-09-07

**Authors:** Chaowen Hou, Yuxin Gao, Hongyu Wang

**Affiliations:** 1 Qilu Institute of Technology, Jinan, China; 2 Shandong University of Engineering and Vocational Technology, Jinan, China; 3 Guangxi Normal University, Guilin, China

**Keywords:** 24-h movement patterns, Alzheimer’s disease, circadian rhythm, exercise timing, glymphatic system, meningeal lymphatics, neurolymphatic clearance, Parkinson’s disease

## Abstract

Although physical activity is consistently associated with lower risks of cognitive decline, dementia, and Parkinson’s disease, most exercise research in neurodegeneration still defines exposure by dose-based variables such as duration, intensity, frequency, step count, and cardiorespiratory fitness. This framework is useful for public-health guidance but biologically incomplete for progressive proteinopathies, in which the temporal accumulation and dissemination of misfolded proteins are central to disease evolution. In this review, we propose a temporal intervention framework in which exercise timing is treated as a mechanistic variable that may influence neurodegenerative biology through glymphatic-meningeal lymphatic clearance, vascular pulsatility, circadian alignment, and sleep architecture. We focus on Alzheimer’s disease and Parkinson’s disease because tau and α-synuclein propagation are disease-relevant processes that may be most amenable to intervention during preclinical or prodromal stages. Morning exercise may act primarily through circadian entrainment, afternoon exercise through vascular-metabolic stimulation, appropriately timed evening or low-intensity activity through sleep-related pathways, and sedentary fragmentation through effects on daytime cerebrovascular dynamics and rest-activity rhythm stability. Although the glymphatic-meningeal lymphatic axis provides a plausible link between timed exercise and extracellular protein handling, current human evidence remains indirect and relies heavily on imaging surrogate markers. Future matched-dose trials should integrate accelerometry, sleep physiology, circadian measures, vascular assessments, neurolymphatic imaging, and disease-specific protein biomarkers, including tau PET, plasma p-tau217, and α-synuclein seed amplification assays. Timed exercise should therefore be viewed as a testable biological perturbation that may clarify whether movement timing influences protein propagation in early neurodegenerative disease, not as a proven disease-modifying treatment.

## 1 Introduction

Exercise is increasingly recognized as a modifiable intervention with applications beyond symptomatic rehabilitation and is consistently associated with lower risks of cognitive decline, dementia, and Parkinson’s disease (Xu et al., 2010; Iso-Markku et al., 2022; Langeskov-Christensen et al., 2024). Nevertheless, the dominant conceptual framework remains largely dose based. In most epidemiological studies and clinical trials, exercise exposure is defined by total activity volume, step count, weekly duration, intensity, frequency, or cardiorespiratory fitness. This approach is useful for public-health recommendations but biologically incomplete for neurodegenerative disease. Alzheimer’s disease (AD) and Parkinson’s disease (PD) are progressive proteinopathies in which the spatiotemporal accumulation of misfolded proteins is closely linked to clinical decline. They are not merely disorders of reduced neuronal resilience. A central unresolved question is whether exercise can influence the spread of tau and α-synuclein pathology in the aging brain.

Recent biomarker studies have sharpened this question. In cognitively intact older adults at risk for Alzheimer’s disease, higher physical activity has been associated with slower amyloid-related inferior temporal tau accumulation and slower cognitive and functional decline; however, this association was not explained by lower amyloid burden. Dose-response analyses further suggested that the association with slower tau accumulation plateaued at moderate activity levels, approximately 5,001–7,500 steps per day (Yau et al., 2025). These findings suggest that the disease-relevant target of exercise may lie downstream of amyloid deposition, closer to tau accumulation, tau spread, and physiological mechanisms governing extracellular protein handling. They do not, however, establish causality. A parallel logic is emerging for α-synuclein in Parkinson’s disease. α-synuclein pathology involves extracellular release, transfer, templated seeding, and intracellular aggregation. If exercise is to be considered a disease-modifying intervention, its effects should be evaluated with biomarkers related to protein propagation and clearance, not only with generic markers of neuroprotection.

The glymphatic-meningeal lymphatic axis provides a mechanistic bridge among exercise, sleep, vascular physiology, and protein handling. In this review, neurolymphatic clearance is an umbrella term for glymphatic cerebrospinal fluid-interstitial fluid (CSF-ISF) exchange, perivascular solute transport, meningeal lymphatic drainage, and related CSF outflow routes; it does not denote one anatomical pathway or a validated human clearance measurement. The glymphatic model proposes that CSF enters the brain through periarterial spaces, exchanges with interstitial fluid in the parenchyma, and helps remove extracellular solutes, including amyloid-β and other protein species (Iliff et al., 2012). This process is closely linked to behavioral state and is influenced by the perivascular localization of astrocytic aquaporin-4 (AQP4). Circadian studies suggest that glymphatic influx and drainage vary across the rest-activity cycle, and experimental studies show that sleep enhances metabolite clearance from the adult brain (Xie et al., 2013; Mestre et al., 2018a; Hablitz et al., 2020). Meningeal lymphatic vessels provide a route through which CSF and macromolecules can drain from the central nervous system to peripheral lymphatic structures (Ahn et al., 2019; Thomas et al., 2019). The term should therefore be used cautiously: animal tracer studies provide the strongest mechanistic evidence, whereas human measurements are imaging-derived surrogates of only part of this network.

Exercise may influence this clearance network through several convergent pathways. Acute and chronic physical activity affect cardiovascular function, arterial pulsatility, cerebrovascular reactivity, autonomic tone, systemic inflammation, sleep quality, and circadian organization. These factors are not incidental to lymphatic physiology; they are plausible regulators of it. A recent human MRI study reported that long-term regular exercise was associated with increased meningeal lymphatic vessel flow and putative glymphatic influx (Yoo et al., 2025). These findings support exercise-related changes in clearance-related imaging physiology, but they do not establish enhanced brain-waste clearance or removal of tau or α-synuclein in humans. This distinction is essential because current human MRI measures are imaging-derived surrogates rather than direct measurements of solute or protein elimination. The next step is therefore to test whether specific exercise prescriptions can alter sleep-coupled neurolymphatic physiology and protein-propagation trajectories in biomarker-defined populations, rather than asking whether exercise generally “improves brain clearance.”

Timing is the missing variable in this model. The biological effects of exercise are unlikely to be constant across the day. Depending on intensity, duration, and proximity to bedtime, evening activity may either support or disrupt slow-wave sleep; morning activity may strengthen circadian entrainment and stabilize rest-activity rhythms; and afternoon activity may provide a vascular-metabolic stimulus during a period of high physiological performance. These differences matter because sleep is not merely a confounder in neurodegeneration research. It may act as a gateway through which exercise-induced vascular and metabolic changes are converted into nocturnal clearance. The observation that disrupted rest-activity rhythms and fragmented daily activity patterns are associated with dementia or preclinical cognitive decline (Haghayegh et al., 2024; Wang et al., 2026) supports the view that 24-h movement organization may capture disease-relevant biology that total activity volume alone cannot.

**FIGURE 1:**
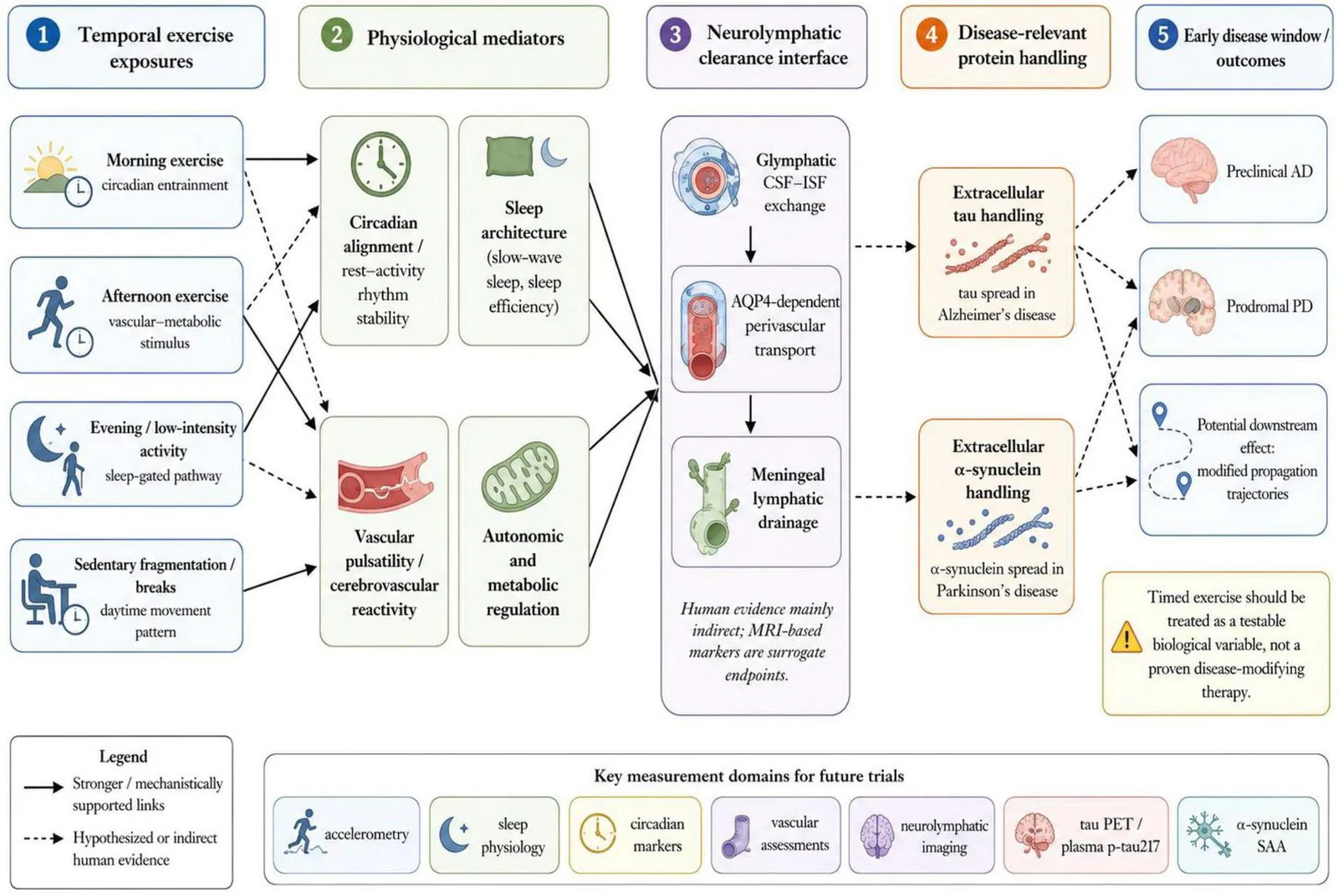
Conceptual framework linking exercise timing, neurolymphatic physiology, and disease-relevant protein handling in early Alzheimer’s disease and Parkinson’s disease. Scheduled exercise is proposed as a biologically meaningful exposure that may influence neurodegenerative proteinopathies through distinct but interconnected physiological pathways. Morning exercise may primarily align the circadian system and stabilize rest-activity rhythms; afternoon exercise may preferentially stimulate vascular and metabolic function; evening or low-intensity activity may influence sleep-related pathways; and sedentary fragmentation or activity breaks may shape daytime movement patterning and cerebrovascular dynamics. These temporal exposures may affect sleep architecture, vascular pulsatility and cerebrovascular reactivity, and autonomic-metabolic regulation, which in turn may modulate the neurolymphatic clearance interface, including glymphatic cerebrospinal fluid-interstitial fluid (CSF-ISF) exchange, AQP4-dependent perivascular transport, and meningeal lymphatic drainage. Changes in neurolymphatic physiology may influence extracellular tau handling in Alzheimer’s disease and extracellular α-synuclein handling in Parkinson’s disease, potentially altering protein-spread trajectories during preclinical or prodromal stages. Solid arrows indicate stronger or more mechanistically supported links, whereas dashed arrows indicate hypothesized pathways or indirect human evidence. Because existing human evidence is mostly indirect, MRI-based neurolymphatic measures should be interpreted as surrogate markers rather than direct measures of clearance. The bottom section highlights key measurement domains for future matched-dose timing studies, including accelerometry, sleep physiology, circadian indicators, vascular assessments, neurolymphatic imaging, tau PET/plasma p-tau217, and α-synuclein seed amplification assays (SAA). Timed exercise should therefore be regarded as a testable biological variable, not as a proven disease-modifying therapy.

This review outlines a framework for timing exercise interventions in neurodegenerative disease (Figure 1). In Figure 1, solid arrows denote stronger or more mechanistically supported links, whereas dashed arrows denote hypothesized links or indirect human evidence; the visual coding is an evidence map, not proof of a complete causal chain. We propose that exercise should be considered both a dose-dependent lifestyle exposure and a time-sensitive neurobiological perturbation. Exercise timing may affect vascular pulsatility, sleep architecture, and circadian alignment, which in turn may regulate clearance-related neurolymphatic physiology. Changes in this physiology could alter extracellular tau and α-synuclein handling and thereby influence early protein spread. The framework is deliberately constrained. We focus on AD and PD because tau and α-synuclein propagation are central to their progression and because both diseases have preclinical or prodromal phases in which protein spread may remain modifiable. We suggest that future trials incorporate exercise timing as a testable biological variable using accelerometry, sleep physiology, neurolymphatic imaging surrogates, and disease-specific protein biomarkers.

## 2 Methods

### 2.1 Review design and scope

**FIGURE 2:**
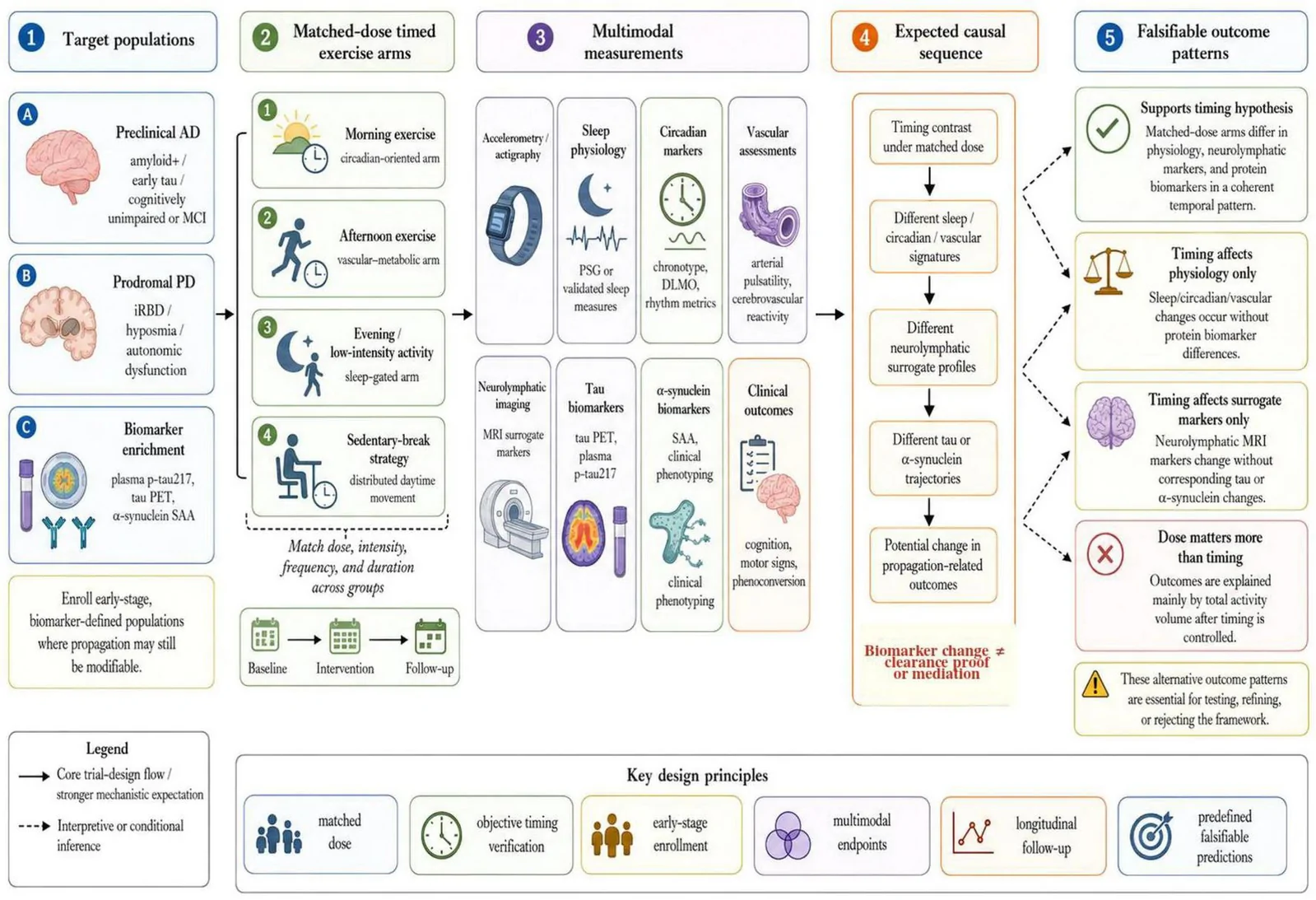
Trial-design framework for testing whether exercise timing influences neurolymphatic physiology and protein propagation in early Alzheimer’s disease and Parkinson’s disease. This figure outlines a biomarker-oriented trial structure for evaluating exercise timing as a mechanistic variable rather than a simple behavioral exposure. Target populations should preferentially include early-stage, biomarker-defined groups in whom protein propagation may still be modifiable, including preclinical Alzheimer’s disease and prodromal Parkinson’s disease. Exercise arms should compare timing strategies under matched prescribed dose, with timing verified objectively and internal load, adherence, light exposure, meals, medication timing, and non-intervention activity recorded. Panel 4 is a hypothesis-generating sequence, not a mediation claim: a fall in plasma p-tau217 or a change in α-synuclein seed-amplification kinetics is not a “smoking gun” for neurolymphatic clearance and may reflect production, release, phosphorylation, redistribution, peripheral kinetics, or assay behavior. The expected sequence must therefore be tested with repeated measurements and convergent sleep, vascular, imaging, protein, and clinical endpoints. The right-hand panel preserves the falsifiable alternatives: timing may affect physiology only, imaging surrogates only, protein biomarkers through a non-clearance pathway, or no outcome after dose is controlled.

This review was designed as a critical, mechanism-oriented narrative review rather than a systematic review or meta-analysis. Its aim was not to estimate a pooled effect of exercise on clinical outcomes, but to develop a testable temporal framework linking exercise timing, sleep and circadian biology, cerebrovascular physiology, glymphatic-meningeal lymphatic function, and protein propagation in Alzheimer’s and Parkinson’s disease. We focused on these two disorders because they have biologically definable early stages, relevant protein pathologies, and measurable biomarkers associated with tau or α-synuclein. The primary question was whether exercise timing should be treated as a core variable that can influence neurolymphatic physiology and protein distribution, rather than as a secondary feature of total exercise dose. The resulting biomarker-oriented trial framework is shown in Figure 2.

### 2.2 Literature search

Literature searches were conducted in PubMed, Web of Science Core Collection, and Google Scholar through 28 February 2026. English-language articles were eligible. Peer-reviewed primary studies and reviews were prioritized; preprints were eligible only when directly relevant and are identified as preliminary, whereas conference abstracts and unpublished reports were excluded. The database-specific strings were: PubMed, [(exercise OR physical activity* OR walking OR sedentary break*) AND (timing OR time-of-day OR circadian OR chronotype OR rest-activity OR 24-h) AND (glymphatic OR meningeal lymphatic OR cerebrospinal fluid OR AQP4 OR vascular pulsatility OR sleep) AND (Alzheimer* OR dementia OR Parkinson* OR synucleinopathy OR tau OR alpha-synuclein)]; Web of Science, TS = [(exercise OR physical activity* OR walking OR sedentary break*) AND (timing OR time-of-day OR circadian OR chronotype OR rest-activity OR 24-h) AND (glymphatic OR meningeal lymphatic OR cerebrospinal fluid OR AQP4 OR vascular pulsatility OR sleep) AND (Alzheimer* OR dementia OR Parkinson* OR synucleinopathy OR tau OR alpha-synuclein)]; and Google Scholar, “exercise timing” glymphatic Alzheimer tau, “physical activity timing” Parkinson synuclein sleep, and “sedentary breaks” cerebrovascular glymphatic. Backward and forward citation tracking was then applied to key articles. Search terms were organized into five domains: exercise and movement timing; sleep and circadian biology; vascular and autonomic physiology; glymphatic-meningeal lymphatic clearance; and disease-specific proteinopathy. A targeted update check was completed on 12 August 2026 to identify directly relevant studies published during revision and to verify the bibliographic metadata of cited studies; newly identified studies were subjected to the same language and eligibility criteria. This update is reported separately from the formal database search because a PRISMA-style screening log was not prospectively maintained for this critical narrative review.

### 2.3 Study selection and prioritization

Studies were selected according to their conceptual and mechanistic relevance, with priority given to primary research examining at least one element of the proposed pathway: exercise timing; sleep regulation; vascular, autonomic, or cerebrospinal fluid physiology; glymphatic or meningeal lymphatic function; tau or α-synuclein handling; or biomarker-defined Alzheimer’s or Parkinson’s disease. Human studies were prioritized when available, particularly cohort studies, clinical trials, accelerometry studies, sleep studies, neuroimaging studies, and biomarker studies. Mechanistic animal studies were used for pathways that remain difficult to measure directly in humans. Because this was a critical narrative review rather than a systematic review, no PRISMA-style screening log was maintained: the number of records screened, duplicates, and study-level reasons for exclusion were not prospectively recorded and cannot be reconstructed reliably. Selection was author-led; no formal duplicate independent screening or validated risk-of-bias workflow was prespecified, and disagreements, when they arose, were resolved by discussion. This limitation is stated explicitly rather than replaced by unsupported screening numbers. Reviews, consensus statements, and methodological papers were used to clarify terminology, disease-stage definitions, biomarker interpretation, and imaging limitations.

### 2.4 Disease-specific focus

For Alzheimer’s disease, this review emphasized studies of amyloid status, tau PET, fluid phosphorylated tau biomarkers, early or preclinical symptoms, physical activity, sleep, and tau accumulation or spread. Particular attention was given to evidence suggesting that physical activity may affect disease-relevant processes downstream of amyloid deposition. For Parkinson’s disease, the review emphasized early and prodromal synucleinopathy, including isolated REM sleep behavior disorder, autonomic dysfunction, sleep disturbance, α-synuclein seed amplification assays, and dopaminergic or neuromelanin-sensitive imaging. Studies in established Parkinson’s disease were considered primarily for evidence on exercise feasibility, intensity, adherence, safety, and biomarker-informed trial design.

### 2.5 Evidence synthesis

Evidence was synthesized qualitatively across five domains. To distinguish causal roles, baseline chronotype, disease stage, vascular risk, habitual sleep, and baseline activity were treated as pre-exposure confounders and candidate effect modifiers; assigned exercise timing was the exposure; post-assignment sleep, circadian, autonomic, and vascular changes were candidate mediators; adherence, completed workload, internal physiological load, light exposure, meals, medication timing, and non-intervention activity were measured co-exposures; and protein and clinical outcomes were downstream endpoints. The resulting directed structure is: timing assignment → sleep/circadian/vascular mediators → imaging-derived neurolymphatic surrogates → disease-specific protein endpoints → clinical outcomes. These roles were used to organize hypotheses, not to claim mediation from temporal sequence alone. The same causal roles are displayed explicitly in Supplementary Figure 1 as a directed acyclic graph (DAG), distinguishing pre-exposure confounding/effect modification from post-assignment mediation and implementation variables.

Third, we examined tau and α-synuclein propagation as disease-specific targets, while distinguishing measurement of disease dynamics from measurement of propagation or clearance. Fourth, morning, afternoon, evening, and sedentary-break interventions were treated as distinct exposures. Clock-time labels were descriptive; the preferred exposure definition was time since habitual wake, time to habitual bedtime, and, where feasible, circadian phase. Sedentary-break interventions were not treated as a low-dose substitute for exercise-session timing. Fifth, we proposed biomarker-based trial-design principles, including matched prescribed dose, completed workload, internal load, adherence, objective timing verification, sleep physiology, circadian markers, vascular assessments, neurolymphatic imaging surrogates, disease-specific protein biomarkers, a prespecified primary estimand, and multiplicity control.

### 2.6 Interpretation of neurolymphatic and proteinopathy biomarkers

Because human glymphatic and meningeal lymphatic measurements remain indirect, all MRI-based indicators of neurolymphatic function were interpreted as surrogate endpoints rather than direct measures of protein clearance. These methods included diffusion tensor imaging, enlarged perivascular spaces, cerebrospinal fluid flow measures, contrast-enhanced meningeal lymphatic imaging, and related imaging approaches. Findings from these measures were interpreted cautiously because they may reflect multiple biological and technical factors, including vascular, anatomical, sleep-related, inflammatory, and acquisition-related influences. Protein biomarkers were also interpreted cautiously. Changes in tau PET, plasma p-tau217, cerebrospinal fluid tau, α-synuclein seed amplification assay metrics, or peripheral phosphorylated α-synuclein do not by themselves prove improved clearance. They may reflect changes in production, release, phosphorylation, aggregation, redistribution, degradation, assay sensitivity, or disease stage. Therefore, this review treats biomarker changes as indicators related to proteinopathy dynamics, not as direct evidence of glymphatic or lymphatic clearance.

### 2.7 Methodological limitations

A quantitative meta-analysis was not performed because of substantial heterogeneity in study populations, disease stages, exercise modalities, timing definitions, sleep measures, imaging approaches, biomarker platforms, and outcome estimands. Narrower quantitative syntheses were also not judged feasible because even within domains the exposure contrast, follow-up interval, biomarker assay, and adjustment set were not sufficiently comparable. We did not apply a validated risk-of-bias scale; instead, Table 1 uses a prespecified qualitative profile based on study design, directness to human disease, causal leverage, consistency, and measurement validity. “Moderate” indicates reasonably consistent evidence with important indirectness or residual confounding; “low to moderate” indicates mixed or predominantly indirect evidence; and “very low/unproven” indicates that the complete causal chain has not been demonstrated in humans. For key human studies, sample size, timing definition, effect estimate and confidence interval when reported, covariate adjustment, and major bias were recorded to qualify the narrative claims rather than to create a pooled estimate. The conclusions should therefore be interpreted as a mechanistic framework rather than as a clinical guideline. Its purpose is to define testable hypotheses and trial requirements, not to imply that timed exercise is an established disease-modifying treatment for Alzheimer’s or Parkinson’s disease. The requested study-level information is now presented explicitly in Supplementary Table 1, including sample size, exposure/timing definition, principal effect estimate and uncertainty when reported, statistical adjustment or analysis strategy, and the main inferential limitation for each key human study.

**TABLE 1:** Evidence map and qualitative evidence profile supporting the timed-exercise-neurolymphatic-proteinopathy framework.

Proposed link in the framework	Main evidence base	Evidence profile	Representative evidence cited in this review	Main limitation	Interpretation for future trials
Higher physical activity is associated with lower risk of cognitive decline, dementia, and Parkinson’s disease	Human epidemiological studies, prospective cohorts, exercise trials	Association: moderate; causal: low	Physical activity and exercise are associated with reduced risk of cognitive decline, dementia, and PD (Xu et al., 2010; Iso-Markku et al., 2022; Langeskov-Christensen et al., 2024)	Mostly observational; residual confounding; effects often defined by total dose rather than timing	Supports exercise as a relevant exposure, but does not establish timing-specific or protein-clearance mechanisms
Physical activity may be associated with slower tau accumulation in preclinical AD	Human longitudinal biomarker studies using activity measures and tau PET	Moderate association; indirect timing evidence	Higher activity was associated with slower amyloid-related inferior temporal tau accumulation and slower clinical decline (Yau et al., 2025)	Association does not prove causality; activity timing was not the primary exposure; amyloid and lifestyle confounding remain possible	Justifies testing exercise against tau-related endpoints in biomarker-defined AD populations
Exercise timing can influence circadian alignment and rest-activity rhythm stability	Human circadian, chronotype, actigraphy, and exercise-timing studies	Moderate association; indirect timing evidence	Rest-activity rhythm disruption is associated with cognitive decline or AD-related biomarkers (van Someren et al., 1996; Haghayegh et al., 2024; Winer et al., 2024; Paget-Blanc et al., 2025; Wang et al., 2026); exercise timing affects circadian and sleep-related physiology (Kim et al., 2023; Tariq et al., 2026)	Few studies are in biomarker-defined AD or PD; timing effects may differ by chronotype, light exposure, meals, medication, and baseline sleep disorder	Timing should be measured objectively and analyzed separately from total activity volume
Sleep state regulates glymphatic or CSF–interstitial fluid exchange	Animal tracer studies, human sleep and CSF-flow imaging studies	Animal mechanism; indirect human evidence	Sleep enhances metabolite clearance and CSF/interstitial fluid exchange; glymphatic function is linked to behavioral state and AQP4 localization (Iliff et al., 2012; Xie et al., 2013; Mestre et al., 2018a; Hablitz et al., 2020)	Strongest mechanistic evidence is preclinical; human measures are indirect and technically heterogeneous	Sleep physiology should be treated as a mechanistic mediator, not merely a confounder
Vascular pulsatility, arterial function, and cerebrovascular dynamics may regulate neurolymphatic flow	Animal mechanistic studies, human cerebrovascular physiology, MRI surrogate studies	Low-moderate; indirect and heterogeneous	Vascular pulsatility and cerebrovascular dynamics are implicated in glymphatic-meningeal lymphatic physiology (Iliff et al., 2012; Xie et al., 2013; Carter et al., 2018; Mestre et al., 2018a; Ahn et al., 2019; Wheeler et al., 2019; Hablitz et al., 2020; Maasakkers et al., 2020; Taylor et al., 2022; Horiuchi et al., 2023; Tallon et al., 2023; Yoo et al., 2025)	Vascular markers do not directly quantify protein clearance; causality remains uncertain	Trials should include vascular endpoints such as blood pressure, arterial stiffness, cerebral blood flow, and cerebrovascular reactivity
Long-term exercise is associated with human MRI markers of meningeal lymphatic flow and putative glymphatic influx	Human MRI study of exercise and clearance-related imaging markers	Low-moderate; imaging surrogate only	Long-term regular exercise was associated with increased meningeal lymphatic vessel flow and putative glymphatic influx (Yoo et al., 2025)	Imaging markers are surrogate endpoints; no direct evidence of tau or α-synuclein removal; cross-sectional or non-definitive causal interpretation	Supports inclusion of neurolymphatic imaging, but not as a standalone proof of disease modification
Meningeal lymphatic vessels provide a route for CNS macromolecule drainage	Animal tracer and lymphatic-manipulation studies; emerging human imaging studies	Moderate animal mechanism; low human directness	Meningeal lymphatics drain CSF and macromolecules to peripheral lymphatic structures (Ahn et al., 2019)	Human functional quantification remains limited; drainage routes and dynamics remain debated	Meningeal lymphatic imaging may be useful, but should be paired with sleep, vascular, and protein biomarkers
Neurolymphatic impairment may contribute to AD-related protein accumulation	Animal AD models, human MRI surrogates, sleep and biomarker studies	Low-moderate; disease-specific evidence sparse	Glymphatic-meningeal lymphatic pathways are linked to clearance of amyloid-β and other solutes (Iliff et al., 2012; Xie et al., 2013; Mestre et al., 2018a; Ahn et al., 2019; Hablitz et al., 2020; Yoo et al., 2025)	Direct human evidence linking altered neurolymphatic flow to reduced tau propagation is lacking	AD trials should avoid claiming “clearance” unless protein endpoints and clearance-related physiology move together
Tau propagation is a disease-relevant target in early AD	Human tau PET, amyloid/tau staging, plasma p-tau217 studies	Strong for disease relevance; low for exercise-timing modulation	Tau and amyloid positivity predict cognitive decline; tau PET and plasma p-tau217 support biomarker-based staging (Sperling et al., 2011; Hanseeuw et al., 2019; Insel et al., 2023; Ashton et al., 2024; Jack et al., 2024; Rissman et al., 2024; Sperling et al., 2024)	These biomarkers measure disease dynamics, not necessarily clearance; timing interventions have not been tested against tau spread	Best AD target population: amyloid-positive individuals with absent, limited, or early tau spread
α-synuclein propagation is a disease-relevant target in prodromal and early PD	α-synuclein SAA studies, iRBD cohorts, skin phosphorylated α-synuclein studies, PD staging frameworks	Strong for disease relevance; low for exercise-timing modulation	iRBD predicts synucleinopathy; α-synuclein SAA and skin p-syn support biological enrichment (Berg et al., 2015; Postuma et al., 2015; Doppler et al., 2017; Heinzel et al., 2019; Postuma et al., 2019; Doppler et al., 2021; Concha-Marambio et al., 2023; Siderowf et al., 2023; Dam et al., 2024; Gibbons et al., 2024; Bernhardt et al., 2025; Coughlin et al., 2025)	SAA positivity does not provide a linear measure of propagation burden; skin p-syn is peripheral and not equivalent to brain spread	Best PD target population: iRBD or other at-risk individuals with α-synuclein biomarker positivity
Exercise improves clinical or functional outcomes in PD and MCI/early AD	Human randomized controlled trials and exercise intervention studies	Moderate	Exercise trials in MCI/AD and PD support feasibility and clinical relevance (Uc et al., 2014; Schenkman et al., 2018; van der Kolk et al., 2019; Johansson et al., 2022; Patterson et al., 2022; Baker et al., 2025; Shadyab et al., 2025)	Most trials are not timing-matched, not biomarker-driven, and do not test protein propagation or neurolymphatic endpoints	Existing exercise trials justify feasibility, but new trials must use matched dose and timing-specific design
Sedentary breaks and morning activity may preserve daytime cerebrovascular dynamics	Human experimental studies of prolonged sitting, walking breaks, and cerebral blood flow	Moderate for vascular physiology; low for proteinopathy outcomes	Morning exercise and walking breaks mitigate sitting-related reductions in cerebral blood flow (Carter et al., 2018; Wheeler et al., 2019; Heiland et al., 2020; Maasakkers et al., 2020; Taylor et al., 2022; Horiuchi et al., 2023; Tallon et al., 2023; Cunha et al., 2025)	Effects are short-term physiological endpoints, not disease-modifying outcomes	Sedentary-break arms may be useful for low-tolerance or vascular-risk participants
Morning, afternoon, evening, and sedentary-break exercise may act through different mechanisms	Human exercise-timing, sleep, circadian, vascular, and cardiometabolic studies	Low to moderate	Morning exercise may support entrainment; afternoon exercise may stimulate vascular-metabolic function; evening exercise may affect sleep depending on intensity and timing (Brito et al., 2018; Brito et al., 2019; Yue et al., 2022; Kim et al., 2023; Sevilla-Lorente et al., 2023; Brito et al., 2024; Tariq et al., 2026)	Disease-specific biomarker evidence is sparse; effects likely depend on chronotype, intensity, medication timing, sleep disorders, and frailty	Future trials should compare timing arms under matched dose, intensity, supervision, and adherence
Timed exercise slows tau or α-synuclein propagation through neurolymphatic clearance	No direct human evidence; hypothesis derived from linked mechanisms	Very low/unproven	The framework integrates exercise, sleep, vascular, neurolymphatic, and proteinopathy evidence (Sperling et al., 2011; Iliff et al., 2012; Xie et al., 2013; Berg et al., 2015; Postuma et al., 2015; Doppler et al., 2017; Mestre et al., 2018a; Ahn et al., 2019; Hanseeuw et al., 2019; Heinzel et al., 2019; Postuma et al., 2019; Hablitz et al., 2020; Doppler et al., 2021; Concha-Marambio et al., 2023; Insel et al., 2023; Siderowf et al., 2023; Ashton et al., 2024; Dam et al., 2024; Gibbons et al., 2024; Jack et al., 2024; Rissman et al., 2024; Sperling et al., 2024; Bernhardt et al., 2025; Coughlin et al., 2025; Yau et al., 2025; Yoo et al., 2025)	The full causal chain has not been demonstrated in humans; biomarkers may reflect production, release, redistribution, degradation, or assay effects rather than clearance	This is the central falsifiable hypothesis; it requires multimodal, matched-dose, biomarker-driven trials

AD, Alzheimer’s disease; PD, Parkinson’s disease; CSF, cerebrospinal fluid; MRI, magnetic resonance imaging; PET, positron emission tomography; AQP4, aquaporin-4; iRBD, isolated REM sleep behavior disorder; SAA, seed amplification assay; p-syn, phosphorylated α-synuclein.

## 3 Findings

### 3.1 Exercise timing and dose in neurodegeneration

#### 3.1.1 Limitations of dose-based exercise prescriptions

Dose-based guidelines have traditionally shaped exercise prescriptions for neurological disorders and aging. Public-health and clinical guidelines typically specify frequency, intensity, duration, and exercise type to improve cardiorespiratory fitness, muscle strength, metabolic health, and functional ability (Garber et al., 2011; Piercy et al., 2018). This model has clear practical value because it translates physical activity into measurable targets, including intensity zones, daily step counts, metabolic equivalents, and minutes per week. Most observational studies and intervention trials in neurodegeneration use the same framework: physical activity exposure is defined by total volume, weekly duration, step count, moderate-to-vigorous activity, sedentary time, or cardiorespiratory fitness. These variables are important but incomplete. A 30-min morning walk, an equivalent evening walk, and brief movement distributed throughout the day can yield similar activity totals while producing different effects on circadian rhythms, vascular physiology, autonomic regulation, and sleep. Dose-based frameworks are therefore necessary but insufficient for understanding exercise as a potential disease-modifying intervention in AD and PD.

Large prospective studies confirm the importance of physical activity volume for dementia prevention. Accelerometer-based analyses of UK Biobank step counts showed that higher daily steps and greater step intensity were associated with lower dementia risk (Del Pozo Cruz et al., 2022). Other accelerometer studies have linked lower physical activity, higher sedentary time, or both with higher risks of dementia or cognitive decline (Raichlen et al., 2023; Zhong et al., 2023). These studies are valuable because they move beyond self-reported activity and provide objective behavioral phenotypes. However, they remain largely dose focused, emphasizing accumulated activity or total sedentary time. They do not resolve whether movement timing, distribution, and rhythmicity independently modify neurodegenerative risk. In other words, total activity level alone does not indicate whether a person’s movement pattern is aligned with the sleep-wake cycle, vascular rhythms, or nocturnal clearance phases.

The limits of dose-only models become clearer when exercise is evaluated against disease-specific biology. Alzheimer’s disease and Parkinson’s disease progress through sequential protein accumulation, network vulnerability, sleep-wake disruption, autonomic dysfunction, and motor impairment. A single cumulative activity measure cannot determine whether physical activity preserves daytime vascular dynamics, occurs before or after circadian disruption, or improves or degrades sleep architecture. It also cannot determine whether exercise is delivered during a disease stage in which tau or α-synuclein propagation remains modifiable. This matters because different biological outcomes imply different clinical interpretations of exercise. The amount, timing, and distribution of activity needed to influence sleep-related glymphatic clearance or extracellular protein handling may differ from what is needed to improve overall cardiovascular health.

Reverse causality is another problem. Reduced physical activity may be both an early marker and a risk factor for neurodegenerative disease. Early Parkinson’s disease can involve subtle motor slowing, fatigue, autonomic symptoms, depression, or sleep disturbance. In early Alzheimer’s disease, apathy, sleep disruption, or circadian fragmentation may alter daily movement before overt cognitive decline. Therefore, the association between low activity and future disease is not automatically causal. Objective activity studies reduce measurement error, but mechanistic interpretation remains essential. To frame exercise as a disease-modifying exposure, future studies must test whether specific activity patterns influence intermediate biological factors such as sleep physiology, cerebrovascular function, glymphatic-meningeal lymphatic markers, and protein biomarkers.

The dose model has also encouraged a false dichotomy between “exercise” and “sedentary behavior.” In reality, the brain exists within a continuous 24-h behavioral cycle that includes sleep, sedentary periods, light-intensity activity, and moderate-to-vigorous activity. These behaviors compete for time and may have distinct biological effects. Structured exercise may not fully offset the neurodegenerative consequences of prolonged inactivity. Total daily movement, sedentary-bout fragmentation, and alignment of activity with endogenous rhythms may shape the vascular and sleep-dependent conditions of brain homeostasis. This reframes dose as one component of a broader temporal exposure; it does not make dose irrelevant.

#### 3.1.2 Exercise is a 24-h rhythmic exposure.

Physical activity is more than an index of energy expenditure. It is also a non-photic time cue that can influence circadian stability, amplitude, and phase. Experimental and clinical studies show that timed exercise can alter melatonin dynamics, shift circadian phase, and interact with chronotype (Buxton et al., 2003; Gabriel and Zierath, 2019; Thomas et al., 2020; Ning et al., 2025). In one controlled intervention, the circadian phase-shifting effect of exercise varied by individual chronotype and exercise timing (Ning et al., 2025). Earlier studies also found that exercise can acutely alter melatonin secretion and phase depending on time of day (Thomas et al., 2020). Because circadian disruption is common in aging, Alzheimer’s disease, and Parkinson’s disease and may be more than a downstream symptom, these findings are directly relevant to neurodegeneration (Leng et al., 2019).

When physical activity is treated as a 24-h rhythmic exposure, the unit of analysis changes. Instead of asking only how many minutes of activity are accumulated each week, the field should ask when activity occurs relative to habitual wake time and bedtime, whether it is aligned with circadian phase, and whether it strengthens or weakens rest-activity rhythms. Morning, afternoon, and evening are therefore descriptive labels rather than biologically equivalent exposure categories. Relevant variables include time since habitual wake, time to bedtime, peak activity timing, intradaily variability, interdaily stability, sedentary-bout duration, daytime napping, nocturnal movement, and activity timing relative to dim-light melatonin onset. Sedentary-break interventions should be analyzed separately from session-timing comparisons.

Research on rest-activity rhythms provides an important link between neurodegeneration and movement science. A 2026 ARIC study found that later peak activity timing and weaker, more fragmented rest-activity rhythms were associated with a higher risk of dementia in older adults (Wang et al., 2026). Other cohort studies have linked weakened rest-activity rhythms to dementia, mild cognitive impairment, and cognitive impairment in older populations (Leng et al., 2019). These findings suggest that movement organization throughout the day contains disease-relevant information that total physical activity volume alone cannot capture, but they do not demonstrate that changing activity timing will prevent dementia. The rest-activity rhythm may be a behavioral indicator of early neurodegenerative burden, autonomic regulation, motor function, sleep quality, and central circadian stability.

Because sleep and exercise are biologically linked, a 24-h perspective is essential. Physical activity affects sleep duration, sleep efficiency, deep sleep, thermoregulation, autonomic balance, and perceived sleep quality. Conversely, insufficient sleep can reduce daily activity, increase perceived exertion, and alter exercise behavior. This reciprocal relationship creates a methodological challenge for conventional exercise research: sleep is often treated as a confounder, although it may be a mediator or even a critical pathway through which exercise influences neurodegeneration. If glymphatic and meningeal lymphatic clearance are most active during sleep or selected rest states, exercise timing may determine whether activity supports or impairs clearance-related physiology. Thus, a high-intensity evening session that disrupts slow-wave sleep may have different consequences from an afternoon session that improves sleep consolidation, even when total moderate-to-vigorous activity is matched.

This framework also challenges the idea that structured exercise is the only relevant behavioral exposure. For older adults or individuals in early disease stages who cannot safely tolerate high-intensity exercise, light activity, frequent breaks from sitting, and a regular daytime activity pattern may be especially useful. Parkinson’s disease can constrain exercise timing and intensity through autonomic dysfunction, orthostatic hypotension, fatigue, freezing of gait, and fall risk. In preclinical AD or MCI, schedule regularity and sleep quality may be more important than maximal intensity. The optimal timing strategy may therefore vary by chronotype, symptom profile, and disease stage. Rather than simply increasing activity volume, a biologically informed intervention should organize daily activity to support circadian stability, vascular regulation, and sleep-dependent clearance.

Recent studies of activity timing and dementia risk reinforce the need to examine temporal patterns rather than total volume alone. In the broader shift toward wearable-based temporal phenotyping, accelerometer-measured physical activity timing has been studied in relation to incident dementia (Ning et al., 2025). These studies remain observational and require cautious interpretation, but they provide empirical support for moving from “how much activity” to “when activity occurs.” The next step is to link temporal activity metrics to mechanistic biomarkers, including sleep architecture, cerebrovascular pulsatility, glymphatic imaging proxies, and protein-specific outcomes. Without such integration, activity timing will remain an epidemiological association rather than a testable neurobiological intervention.

#### 3.1.3 A temporal intervention hypothesis for proteinopathy

We propose that the effect of exercise on neurodegenerative proteinopathies depends not only on dose but also on the biological state in which that dose is delivered. The testable sequence is: assigned timing (A) → sleep, circadian, vascular, and autonomic mediators (M1) → imaging-derived neurolymphatic surrogates (M2) → disease-specific protein dynamics (Y1) → clinical progression (Y2). Each arrow is a separate hypothesis. A difference in a downstream biomarker does not, by itself, establish that either M1 or M2 mediated the effect.

This hypothesis is intentionally more specific than the general claim that exercise is neuroprotective. Neurotrophic, anti-inflammatory, mitochondrial, metabolic, and vascular mechanisms all matter, but the temporal framework asks when these mechanisms are activated and how they interact with sleep and clearance physiology. Exercise-induced increases in cerebral blood flow, vascular shear stress, autonomic modulation, or metabolic signaling may have different consequences depending on whether they occur during a circadian phase that supports subsequent sleep and clearance. Timing does not replace dose; it may determine which biological pathway a given dose preferentially engages.

For AD, this framework predicts that timed exercise may have stronger effects on tau-related processes than on amyloid burden. This prediction is consistent with biomarker data linking physical activity to slower cognitive and functional decline and lower tau accumulation in preclinical Alzheimer’s disease (Yau et al., 2025). The temporal model suggests that exercise may be most relevant when it improves sleep-related clearance or reduces extracellular tau persistence and network spread. Suitable trial participants would therefore include amyloid-positive individuals with minimal or spatially limited tau pathology, rather than patients with advanced dementia in whom tau spread and neurodegeneration are already extensive.

For Parkinson’s disease, the framework predicts that timed exercise may be most relevant in individuals with prodromal sleep or autonomic phenotypes. Isolated REM sleep behavior disorder, hyposmia, constipation, autonomic symptoms, and α-synuclein SAA positivity can indicate α-synuclein pathology before overt parkinsonism. If sleep architecture and circadian regulation influence clearance biology, timed exercise may affect α-synuclein propagation before clinical expression. This claim remains unproven, but it is more mechanistically precise than asking only whether exercise improves motor symptoms after diagnosis. The hypothesis is also falsifiable: timing may change sleep or neurolymphatic imaging markers without altering tau or α-synuclein biomarkers; protein biomarkers may change without corresponding clearance-related changes; or total activity volume may predict outcomes better than timing after adjustment for sleep, circadian phase, and disease stage. These outcomes would refine or reject the model rather than merely weaken it.

Temporal intervention models require specific trial structures. The primary comparison should be between exercise arms with matched prescribed external dose, not between exercise and education, because a usual-activity arm does not isolate timing. The primary estimand should be the intention-to-treat effect of assigned timing on one prespecified primary endpoint under matched prescribed dose; completed workload, internal physiological load, adherence, and non-intervention activity should be reported separately and used in sensitivity or per-protocol analyses. A short proof-of-mechanism trial should select one proximal endpoint, whereas a longer disease-modification trial should select one disease-specific endpoint. Secondary outcomes should be ordered prospectively from sleep/circadian physiology to vascular measures, imaging-derived surrogates, protein biomarkers, and clinical outcomes.

### 3.2 The glymphatic-meningeal lymphatic axis as a modifiable clearance interface

#### 3.2.1 Neurolymphatic clearance anatomy and physiology

Although the brain parenchyma lacks conventional lymphatic vessels, the central nervous system must continuously remove protein species, metabolic waste products, and extracellular solutes from a densely packed tissue environment. The glymphatic model (Iliff et al., 2012) describes cerebrospinal fluid (CSF) entering the brain through periarterial spaces, exchanging with interstitial fluid (ISF) within the parenchyma, and facilitating interstitial solute clearance toward perivenous and meningeal drainage pathways. In this framework, astrocytic endfeet enriched in aquaporin-4 (AQP4) support periarterial CSF influx, CSF-ISF exchange, and solute efflux, all anatomically linked to the cerebral vasculature. The pathway is therefore a dynamic neurovascular-glial clearance route rather than passive plumbing, and its efficiency depends on extracellular-space geometry, astrocytic water transport, vascular motion, and behavioral state.

The discovery of meningeal lymphatic vessels substantially expanded this model. Functional lymphatic vessels lining the dural sinuses in mice expressed lymphatic endothelial markers and connected to deep cervical lymph nodes (Louveau et al., 2015). A subsequent study identified a dural lymphatic vascular system capable of draining CSF and macromolecules to extracranial lymphatic structures after uptake from the adjacent subarachnoid space (Aspelund et al., 2015). Later work supported the view that meningeal lymphatics provide a downstream pathway through which CSF, immune cells, and macromolecules leave the cranial compartment (Ahn et al., 2019). Together, these findings shifted the brain-clearance paradigm from a purely parenchymal pathway to an integrated glymphatic-meningeal lymphatic axis. Louveau et al. and Aspelund et al. independently identified functional dural lymphatic vessels, whereas Ahn et al. emphasized skull-base vessels as important CSF drainage routes (Aspelund et al., 2015; Louveau et al., 2015; Ahn et al., 2019).

In this review, “neurolymphatic clearance” refers to glymphatic CSF-ISF exchange, perivascular solute transport, meningeal lymphatic drainage, and related CSF outflow routes. This terminology is useful because exercise is unlikely to affect only one isolated anatomical compartment. Changes in arterial pulsatility can influence perivascular CSF influx; changes in sleep architecture can alter extracellular space and solute movement; and changes in meningeal lymphatic function may influence downstream drainage. These processes are physiologically connected but experimentally distinguishable. Brain clearance capacity should therefore be treated as a network property rather than as the function of a single pathway.

This distinction matters in neurodegenerative disease. The progression of AD and PD depends not only on protein production or aggregation but also on the movement, persistence, and clearance of extracellular protein species. If extracellular tau or α-synuclein seeds remain in the interstitial compartment for prolonged periods, they may interact with vulnerable neural networks. The glymphatic-meningeal lymphatic axis is conceptually attractive because it links protein clearance to modifiable systemic variables, including sleep, vascular function, arterial pulsatility, inflammation, autonomic tone, and physical activity. That attractiveness also creates a risk of overinterpretation. The existence of neurolymphatic pathways does not mean that exercise alters tau or α-synuclein spread. It defines a plausible mechanistic interface that must be tested with disease-specific biomarkers.

Aging increases the relevance of this interface. In mice, aging is associated with reduced CSF-ISF exchange, impaired interstitial amyloid-β clearance, reduced arterial wall pulsatility, and loss of perivascular AQP4 polarization (Kress et al., 2014). Human neuropathological data link altered perivascular AQP4 localization with age, cognition, and AD pathology (Zeppenfeld et al., 2017). These findings suggest that impaired solute transport, astrocytic endfoot disruption, and vascular aging may contribute to clearance failure. Experimental models further show that meningeal lymphatic dysfunction can exacerbate AD-like pathology and age-related cognitive decline (Da Mesquita et al., 2018). Exercise should therefore be investigated not only as a general enhancer of cognitive resilience but also as a potential moderator of clearance systems that deteriorate with age and proteinopathy burden. Zeppenfeld et al. linked loss of perivascular AQP4 localization to aging and AD-related pathology in human brains, whereas Kress et al. found age-related reductions in CSF-ISF exchange and amyloid-β clearance in mice (Kress et al., 2014; Zeppenfeld et al., 2017).

#### 3.2.2 Sleep-related flow, AQP4 polarization, and vascular pulsatility

Vascular pulsatility, AQP4 polarization, and sleep-dependent flow are especially important for linking exercise to neurolymphatic clearance. These features are interdependent. Sleep influences neural activity, extracellular volume, and CSF-ISF exchange; astrocytic endfeet regulate water exchange at the gliovascular interface; and arterial pulsations can drive perivascular CSF movement. Together, they provide a mechanistic basis for how exercise-induced cardiovascular and sleep effects may influence protein clearance.

Vascular pulsatility is a key potential driver of lymphatic transport. In mice, cerebral arterial pulsation drives paravascular CSF-ISF exchange, and suppressing arterial pulsatility reduces glymphatic influx (Iliff et al., 2013). Subsequent imaging and modeling studies showed that CSF flow is pulsatile and linked to the cardiac cycle, with arterial wall motion closely matching CSF movement in perivascular spaces (Mestre et al., 2018b). More recent work linked neurovascular coupling with glymphatic dynamics: functional hyperemia increased periarterial CSF influx velocity and improved glymphatic clearance in the activated cortical hemisphere (Holstein-Rønsbo et al., 2023). These findings indicate that vascular dynamics and brain activity may cooperate to regulate waste removal, not only metabolic supply. Iliff et al. and Mestre et al. provided strong evidence that arterial pulsatility influences paravascular CSF movement, and Holstein-Rønsbo et al. showed that neurovascular coupling can accelerate glymphatic influx and clearance in mice (Iliff et al., 2013; Mestre et al., 2018b; Holstein-Rønsbo et al., 2023).

AQP4 provides the second mechanistic link. AQP4 is enriched in astrocytic endfeet surrounding cerebral vessels and is thought to support water movement between perivascular spaces and the interstitium. Experimental evidence shows reduced glymphatic solute transport when AQP4 is disrupted (Mestre et al., 2018a). In aging and AD-related pathology, not only AQP4 abundance but also its polarized localization at perivascular astrocytic endfeet can be altered (Kress et al., 2014; Zeppenfeld et al., 2017). This distinction is critical because water transport at the gliovascular interface depends on whether AQP4 remains concentrated in the perivascular endfoot domain. Disruption of perivascular AQP4 localization in mice reduces glymphatic exchange and promotes amyloid-β plaque formation (Simon et al., 2022). Astrocytic endfoot organization is therefore not simply a structural feature; it may determine whether clearance pathways remain functional under conditions of aging and proteinopathy.

Sleep is the third major regulator. Experimental evidence shows that sleep enhances metabolite clearance from the adult brain, accompanied by increased convective exchange between CSF and ISF and expansion of interstitial space (Xie et al., 2013). Circadian studies also show that glymphatic influx and lymphatic drainage vary across the rest-activity cycle (Hablitz et al., 2020). Neurolymphatic clearance therefore appears temporally gated: it changes with behavioral state and possibly circadian phase. This is crucial for exercise. Exercise that fragments or suppresses slow-wave sleep may reduce clearance despite increasing total physical activity, whereas exercise that improves subsequent sleep architecture may support clearance-related physiology.

Because glymphatic flow is sleep dependent, simple exercise recommendations are insufficient. A high-intensity session too close to bedtime may increase arousal, sympathetic tone, or core body temperature and thereby disrupt sleep in vulnerable individuals. Conversely, well-timed exercise may improve slow-wave sleep, stabilize rest-activity rhythms, and enhance sleep efficiency. The same exercise dose may therefore have different effects on clearance depending on timing. The field should move from an exposure model based solely on activity volume toward one that incorporates timing relative to sleep and circadian phase.

In humans, these mechanisms remain hypothetical rather than proven. Most direct evidence for vascular pulsatility, AQP4 localization, and sleep-dependent glymphatic flow comes from animal studies using tracers, microscopy, or controlled physiological manipulation. Human studies are necessarily more indirect. Nevertheless, the convergence of animal physiology, human sleep research, and emerging imaging markers provides a plausible mechanistic explanation: exercise may influence neurolymphatic clearance by altering cardiovascular and sleep-related drivers of CSF-ISF exchange. The burden of evidence is now to show whether these changes are large, specific, and durable enough to influence tau or α-synuclein spread.

#### 3.2.3 Exercise as a regulator of clearance pathways

Exercise is uniquely positioned to regulate neurolymphatic clearance because it affects many physiological variables that also control lymphatic drainage and CSF-ISF exchange. Acute exercise increases heart rate, cardiac output, blood-pressure oscillations, respiratory depth, cerebral perfusion demand, and neurovascular coupling. Chronic exercise improves endothelial function, arterial compliance, autonomic balance, metabolic flexibility, systemic inflammation, and sleep quality. These adaptations may influence neurolymphatic clearance through vascular pulsatility, cerebrovascular reactivity, AQP4 organization, and sleep-dependent flow. Exercise clearly affects the brain, but that observation is too broad. The key question is whether exercise affects clearance pathways in ways that are relevant to protein propagation. Canonichesi et al. (2025) further situate exercise-related sleep modulation within synucleinopathies; this supports an upstream sleep-neurovascular-glial interface, but it should not be interpreted as direct evidence that exercise increases human glymphatic flux or α-synuclein removal.

Animal studies provide the most direct evidence. Voluntary wheel running in aged mice reduced amyloid-β burden, increased astrocytic AQP4 expression and polarization, and accelerated glymphatic clearance (He et al., 2017). In an AD mouse model, AQP4 deficiency eliminated the beneficial effects of voluntary exercise, suggesting that some exercise-induced neuroprotection may require AQP4-dependent glymphatic transport (Liu et al., 2022). Swimming training improved learning and memory in APP/PS1 mice by increasing CSF-ISF exchange, promoting AQP4 polarization, and reducing hippocampal amyloid-β deposition (Liang et al., 2025). Together, these findings support the view that exercise can influence glymphatic-associated biology in animal models, particularly through AQP4-linked mechanisms. He et al. reported that voluntary running increased AQP4 polarization and glymphatic clearance in aged mice; later work suggested that AQP4 disruption may reduce the benefits of exercise in AD models (He et al., 2017; Liu et al., 2022; Liang et al., 2025).

Translational interpretation must be constrained. Most animal exercise-glymphatic studies focus on amyloid-β or cognitive function rather than tau or α-synuclein spread. Many use young or genetically modified animals under conditions that do not fully represent human aging, vascular disease, fragmented sleep, or mixed pathology. Exercise modality, duration, intensity, and timing also vary widely. Swimming, forced treadmill exercise, and voluntary running differ in stress load, thermoregulation, autonomic effects, and circadian consequences. Animal data support biological plausibility, but they do not define an optimal exercise regimen for human proteinopathies.

Human evidence is beginning to emerge but remains limited. A recent MRI study in healthy adults associated long-term regular exercise with increased meningeal lymphatic vessel flow and putative glymphatic influx (Yoo et al., 2025). This study is important because it shows that exercise-related differences in human clearance-related imaging physiology are measurable. However, it did not establish whether exercise timing, sleep architecture, or circadian phase mediated the imaging differences, and it did not directly measure glymphatic flux or removal of tau or α-synuclein. Its main contribution is therefore methodological feasibility, not proof of enhanced protein clearance in humans.

Exercise may influence neurolymphatic clearance through several non-exclusive pathways. First, by improving cardiovascular function and cerebrovascular reactivity, exercise may strengthen hemodynamic forces that support perivascular CSF movement. Second, by improving sleep consistency and slow-wave sleep, exercise may lengthen or deepen clearance-permissive brain states. Third, by reducing vascular risk factors such as hypertension, insulin resistance, and systemic inflammation, exercise may help preserve perivascular spaces and astrocytic endfeet. Fourth, by strengthening rest-activity rhythms, exercise may synchronize clearance processes with endogenous circadian timing. Fifth, by interrupting prolonged inactivity, exercise or activity breaks may prevent low-stimulation vascular states that impair cerebrovascular regulation.

All five mechanisms are temporal. Acute exercise may transiently increase vascular pulsatility and neurovascular coupling, but the effect on clearance may depend on whether restorative or disruptive sleep follows the session. Regularity and circadian alignment may determine how much chronic exercise benefits vascular health. Sedentary breaks may not improve fitness substantially, but they can repeatedly activate autonomic and vascular systems across the day. These possibilities support studying exercise timing as a mechanistic variable that activates different clearance-related pathways, rather than as a matter of preference.

The strongest current claim is limited but important: exercise is a plausible regulator of the glymphatic-meningeal lymphatic axis, although the relevant human pathway has not yet been established. A comprehensive test would require simultaneous measurement of exercise timing, activity distribution, sleep architecture, vascular pulsatility, glymphatic or meningeal lymphatic imaging markers, and disease-specific protein biomarkers. Without this integration, the field risks reducing complex temporal physiology to the oversimplified claim that exercise improves brain-waste clearance.

#### 3.2.4 Methodological cautions and human imaging markers

The major obstacle to incorporating glymphatic biology into exercise trials is measurement. Intracisternal tracers, two-photon microscopy, fluorescent dextrans, dynamic imaging, and post-mortem tissue quantification can be used to study glymphatic function in animals, but these methods are invasive, impractical, or unethical in humans. Human studies therefore depend on indirect imaging markers that capture only parts of the neurolymphatic system. These markers are useful, but they should not be treated as direct measurements of glymphatic flux.

DTI-ALPS, or diffusion tensor imaging analysis along the perivascular space, is one of the most widely used non-invasive approaches. The original DTI-ALPS method estimated water diffusivity along medullary perivascular spaces and reported reduced ALPS-index values in AD patients, which were interpreted as impaired glymphatic activity (Taoka et al., 2017). DTI-ALPS has since been applied across many neurological disorders. Its advantages include non-invasiveness, feasibility in large cohorts, and use of standard diffusion MRI. Its limitations are equally important: the ALPS index is influenced by white-matter microstructure, vascular risk, regional anatomy, diffusion acquisition parameters, region-of-interest placement, and image-processing choices. DTI-ALPS should therefore be viewed as a diffusion-based surrogate for perivascular water movement, not as a precise measure of CSF-ISF exchange.

Contrast-enhanced MRI offers a more direct but more invasive approach. Intrathecal gadolinium-enhanced MRI can track tracer distribution and clearance over time and has been proposed as a method for assessing glymphatic function in the human brain (Eide and Ringstad, 2015). Although this approach has provided important insights into CSF tracer dynamics, it requires lumbar puncture and off-label intrathecal contrast administration and is therefore unsuitable for large-scale prevention trials in healthy or preclinical populations. Intravenous dynamic contrast-enhanced MRI and delayed post-contrast imaging can reveal aspects of meningeal lymphatic vessels or glymphatic-meningeal lymphatic drainage, but interpretation is complicated by blood-brain barrier permeability, vascular enhancement, contrast timing, and compartmental modeling. Eide et al. proposed intrathecal gadolinium MRI as a possible human glymphatic assessment method, whereas subsequent reviews have emphasized the methodological heterogeneity of MRI-based human glymphatic and meningeal lymphatic measures (Eide and Ringstad, 2015; Ringstad and Eide, 2024).

Meningeal lymphatic imaging is promising but still emerging. High-resolution contrast-enhanced MRI has visualized candidate meningeal lymphatic vessels in healthy adults and described their morphology (Patel et al., 2023). Other clinically accessible MRI studies have documented age-related impairment of the glymphatic pathway and putative meningeal lymphatic vessels in humans (Zhou et al., 2020). These findings suggest that meningeal lymphatic structures and functional correlates can be studied in living humans. However, segmentation is technically difficult. Enhancement must be distinguished from nearby venous structures, dura, vascular leakage, or non-lymphatic contrast patterns, and inter-rater variability and protocol differences limit cross-study comparability.

Perivascular-space burden is another commonly used imaging marker. Structural MRI can identify enlarged perivascular spaces, which have been associated with aging, small-vessel disease, neurodegenerative disease, and cognitive decline. Because perivascular spaces are proposed conduits for CSF movement, they are anatomically relevant to glymphatic theory. However, enlarged perivascular spaces do not map directly onto increased or decreased glymphatic flow. They may reflect small-vessel disease, inflammation, atrophy, blood-brain barrier dysfunction, vascular stiffness, or impaired drainage. Perivascular-space burden is unlikely to be sensitive enough as a short-term functional endpoint, although it may be useful as a structural vulnerability marker in exercise trials.

A further complication is that human imaging markers may not measure the same biological process. DTI-ALPS, perivascular-space volume, intrathecal contrast clearance, meningeal lymphatic enhancement, CSF-flow MRI, and arterial pulsatility indices reflect different compartments and timescales, yet they are often grouped under the label “glymphatic imaging.” Disagreement among markers should not be dismissed as noise. The neurolymphatic system may be compartmentalized, with different components affected by different diseases or interventions. Recent work on DTI-ALPS interpretation explicitly warns against treating it as a pure glymphatic readout because it may also reflect white-matter change and vascular dysfunction (Satpathi et al., 2025).

The methodological implications for timed-exercise trials are direct. A single imaging marker is insufficient. Robust designs should combine accelerometry for activity timing, polysomnography or actigraphy for sleep, vascular measures such as arterial pulsatility and cerebrovascular reactivity, MRI-based neurolymphatic surrogates, and disease-specific protein biomarkers such as tau PET, plasma p-tau217, or α-synuclein seed amplification assays. The critical test is whether activity timing produces coordinated changes across sleep, vascular, clearance-related, and proteinopathy endpoints, not whether exercise alters one glymphatic marker.

The field should define falsifiable expectations. Morning or afternoon exercise that improves vascular pulsatility and sleep but does not change neurolymphatic imaging markers would weaken the clearance hypothesis. Neurolymphatic marker changes without corresponding tau or α-synuclein biomarker changes would suggest that improved clearance-related physiology may not be sufficient to alter proteinopathy. Protein biomarker changes without sleep or clearance-related marker changes would require alternative explanations, including reduced production, altered phosphorylation, reduced release, or biomarker redistribution. A rigorous argument cannot assume that glymphatic clearance explains all exercise benefits; it must specify evidence that would support, refine, or contradict the model.

In summary, the glymphatic-meningeal lymphatic axis is a biologically plausible and increasingly quantifiable clearance system that could link exercise timing to neurodegenerative protein spread. Animal studies and early human imaging support its potential modifiability, but its role in human Alzheimer’s disease and Parkinson’s disease remains unresolved. The appropriate response is neither overstatement nor dismissal. The axis should be treated as a testable mechanistic interface requiring multimodal imaging, vascular physiology, sleep and circadian phenotyping, temporally resolved exercise measurement, and protein-specific outcomes.

### 3.3 Protein propagation as a disease-relevant target: tau in Alzheimer’s disease and α-synuclein in Parkinson’s disease

#### 3.3.1 Protein propagation versus general neuroprotection

A major limitation of the exercise literature in neurodegenerative disease is that it often frames benefit as generic neuroprotection. Exercise is commonly discussed in relation to neurotrophic signaling, mitochondrial function, synaptic plasticity, inflammation, oxidative stress, insulin sensitivity, and vascular health. These mechanisms are biologically important but insufficiently disease specific. AD and PD are not distinguished only by reduced neuronal resilience. They are progressive proteinopathies in which misfolded proteins accumulate in anatomically patterned, temporally ordered, and network-constrained ways. The disease-modification question is therefore whether exercise can alter the conditions that allow pathogenic proteins to accumulate, persist, spread, and drive downstream neurodegeneration, not merely whether it improves general brain health (Goedert et al., 2017).

The concept of protein propagation has reshaped how neurodegenerative progression is understood. Experiments show that aggregated tau and α-synuclein can display prion-like behavior through cell-to-cell transfer, templated misfolding, and recruitment of endogenous protein (Goedert et al., 2017). This does not mean that AD or PD are infectious prion diseases in the classical sense. Rather, pathogenic protein assemblies may behave as self-amplifying biological agents whose spread is constrained by cellular susceptibility, neuronal connectivity, and clearance capacity. Disease progression can therefore be understood as a balance among protein synthesis, release, aggregation, intercellular transfer, degradation, and extracellular clearance.

In Alzheimer’s disease, tau is especially important because its accumulation more closely tracks clinical progression than amyloid-β once symptoms emerge. According to Braak staging, neurofibrillary tau pathology begins in limbic and transentorhinal regions and then progresses to association neocortex in a predictable anatomical sequence (Braak and Braak, 1991). Contemporary biomarker frameworks still recognize amyloid-β, tau, and neurodegeneration as fundamental components of AD biology, but growing evidence indicates that tau accumulation and spread are more closely linked to cognitive decline than amyloid burden alone (Jack et al., 2018; Ossenkoppele et al., 2022b). Tau PET studies further show that tau distribution is not random: it varies across individuals, follows network architecture, and predicts future cognitive decline (Hoenig et al., 2018; Franzmeier et al., 2019; Franzmeier et al., 2020; Vogel et al., 2020; Biel et al., 2021; Vogel et al., 2021; Lee et al., 2022; Ossenkoppele et al., 2022a). Amyloid-β can accelerate or amplify tau spread, whereas neuronal communication pathways partly constrain it (Vogel et al., 2020; Lee et al., 2022).

α-synuclein propagation provides a related but distinct target in Parkinson’s disease. Lewy bodies and Lewy neurites consist largely of α-synuclein aggregates. Exogenous α-synuclein fibrils can induce endogenous α-synuclein aggregation and produce Parkinson-like pathology (Luk et al., 2012). Mechanistic studies suggest that receptor-mediated uptake pathways, including LAG3-dependent mechanisms, may contribute to pathological α-synuclein transmission (Mao et al., 2016). *In vitro* and *in vivo* models indicate that α-synuclein pathology is a dynamic process involving release, uptake, templating, and spread across vulnerable circuits, rather than a static intracellular deposit (Luk et al., 2012; Mao et al., 2016).

This propagation-centered perspective changes how exercise should be evaluated. If exercise is assessed only by cognitive scores, motor scales, or global brain volume, mechanistic interpretation remains weak. These endpoints can improve through symptomatic compensation, mood, sleep, vascular function, or practice effects without altering proteinopathy. A disease-modification framework should test whether exercise affects intermediate biological steps such as extracellular tau or α-synuclein availability, sleep-dependent protein dynamics, glymphatic-meningeal lymphatic clearance, tau PET accumulation, α-synuclein seeding activity, or the transition from prodromal synucleinopathy to clinically manifest disease. Timed exercise should meet this higher standard before being discussed as more than supportive care.

Protein propagation also strengthens the case for timing. Propagation is not equally modifiable at every disease stage. Once tau or α-synuclein pathology is widespread and accompanied by neuronal loss, glial dysfunction, sleep disruption, and vascular impairment, behavioral interventions may have limited capacity to alter the underlying biology. Preclinical AD and prodromal PD offer more suitable windows because protein pathology is detectable but not yet anatomically saturated. Timed exercise should therefore be tested in populations where propagation is active, measurable, and potentially modifiable, rather than only in late-stage disease.

#### 3.3.2 Alzheimer’s disease: clearance-sensitive tau spread

Although amyloid-β remains an important upstream pathology in AD, tau is the more relevant target for a temporal exercise framework. Amyloid deposition often begins years before symptoms and may plateau before substantial cognitive decline. Tau, by contrast, accumulates in a spatial pattern more closely linked to clinical impairment and neurodegeneration. Tau PET studies show that higher tau burden, especially in temporal and association cortices, can stage disease progression *in vivo* and predict future cognitive decline (Biel et al., 2021; Vogel et al., 2021; Ossenkoppele et al., 2022a). The high future-decline risk among cognitively normal individuals who are positive for both amyloid and tau supports the idea that tau marks a transition from pathological risk to clinically meaningful progression (Ossenkoppele et al., 2022a).

Tau spread cannot be explained solely by physical proximity or local vulnerability. Network-based studies suggest that structural and functional connectivity influence tau accumulation and distribution. Hoenig et al. described tau distribution networks in AD, and Franzmeier and colleagues showed that functional connectivity is associated with tau levels and subsequent tau accumulation (Hoenig et al., 2018; Franzmeier et al., 2019, 2020). Vogel et al. found that amyloid-β burden increased tau beyond what connectivity alone predicted and that epidemic-spreading models based on neuronal communication pathways explained a substantial portion of the spatial tau pattern in humans (Vogel et al., 2020). These findings elevate tau propagation from a post-mortem pathological concept to a measurable systems-level process.

A clearance-sensitive model of tau propagation is biologically plausible because tau is not exclusively intracellular. Extracellular tau can seed aggregation, neuronal activity can influence extracellular tau release, and extracellular or CSF tau species can be transported through perivascular and meningeal lymphatic pathways. Sleep-wake studies support this link. In a mouse seeding model, sleep deprivation increased ISF and CSF tau levels and facilitated tau pathology spread (Holth et al., 2019). Holth et al. showed that brain interstitial fluid tau in mice and CSF tau in humans vary with the sleep-wake cycle. Human sleep deprivation also alters CSF tau and phosphorylated tau species, indicating that sleep loss affects tau kinetics, although the mechanisms may include altered release, phosphorylation, clearance, or compartmental redistribution (Barthélemy et al., 2020).

The glymphatic-meningeal lymphatic axis may contribute to extracellular tau clearance. Patel et al. showed that dural lymphatics regulate clearance of extracellular tau from the central nervous system (Patel et al., 2019). Harrison et al. reported reduced glymphatic function and tau clearance in a mouse model of tauopathy, with regional differences in glymphatic exchange corresponding to tau accumulation. Ishida et al. showed that AQP4-dependent glymphatic function helps remove extracellular tau from the brain to deep cervical lymph nodes and CSF; in a P301S tau model, AQP4 deletion increased phosphorylated tau deposition and neurodegeneration (Ishida et al., 2022). Long-term glymphatic inhibition has also been shown in an Alzheimer’s disease model to worsen tau aggregation and transhemispheric propagation. Although mainly preclinical, these studies support a mechanistic link between impaired clearance and tau propagation.

These data do not show that exercise slows tau propagation by improving glymphatic clearance. Rather, they show that tau propagation is a disease-relevant process that may be sensitive to neurolymphatic clearance, sleep-wake state, and extracellular protein handling. Exercise matters because it can influence upstream clearance regulators, including sleep architecture, arterial pulsatility, cerebrovascular reactivity, inflammation, and rest-activity rhythm stability. The most plausible hypothesis is therefore that timed exercise may indirectly influence tau accumulation by improving physiological conditions that reduce extracellular tau persistence in vulnerable networks.

Recent biomarker findings linking physical activity to slower tau accumulation in preclinical AD support this interpretation (Yau et al., 2025). The key point is not that exercise removes established tau deposits. It may instead slow accumulation of extracellular tau seeds, alter sleep-dependent tau kinetics, improve downstream clearance conditions, or reduce activity patterns that promote propagation. These mechanisms would not necessarily produce large changes in amyloid PET. They may appear instead as improved sleep physiology, lower plasma or CSF p-tau217 trajectories, slower tau PET accumulation, or altered neurolymphatic imaging markers.

Amyloid burden should therefore not be the only endpoint in preclinical AD trials. Amyloid-positive individuals who are tau-negative or tau-limited are a more biologically appropriate target population. The relevant question is whether timed exercise can slow progression from localized tau pathology to wider neocortical spread. Sleep architecture, glymphatic imaging surrogates, plasma p-tau217, and tau PET should be assessed together. A trial should not be considered negative merely because exercise affects tau accumulation without changing amyloid burden. The relevant mechanism may lie downstream of amyloid, in tau propagation, clearance, or sleep-dependent protein dynamics.

#### 3.3.3 Parkinson’s disease: prodromal sleep phenotypes and α-synuclein propagation

PD provides a complementary setting for testing the temporal intervention framework. Clinically, Parkinson’s disease is defined by motor features, but its biological progression can begin years earlier with prodromal sleep, autonomic, olfactory, and gastrointestinal symptoms. α-synuclein pathology is central to the transition from prodromal to clinical disease. Experiments show that preformed α-synuclein fibrils can initiate endogenous α-synuclein aggregation, spread through anatomically connected regions, and cause progressive dopaminergic neurodegeneration and motor impairment in non-transgenic mice (Luk et al., 2012). These findings underpin current models of synucleinopathy propagation.

α-synuclein propagation involves multiple mechanisms, including extracellular release, uptake, endosomal trafficking, templated seeding, and impaired degradation. LAG3 can bind pathological α-synuclein and promote neuron-to-neuron transmission and toxicity (Mao et al., 2016). Heparan sulfate proteoglycans, extracellular vesicles, tunneling nanotubes, lysosomal dysfunction, and impaired autophagy are also implicated. These pathways show that α-synuclein spread is not merely an anatomical phenomenon. It is shaped by cellular uptake mechanisms, extracellular residence time, proteostatic capacity, and clearance-system efficiency.

Lymphatic pathways may contribute to α-synuclein handling. In PD mouse models, reduced AQP4 expression worsened dopaminergic neuronal loss and α-synuclein pathology, possibly through impaired glymphatic clearance (Cui et al., 2021). Zhang et al. reported a relationship between parenchymal α-synuclein and the AQP4-mediated glymphatic system, suggesting that α-synuclein accumulation and glymphatic dysfunction may reinforce each other (Zhang et al., 2023). Braun et al. found that AQP4 mislocalization slows glymphatic α-synuclein clearance and proposed that disruption of sleep-active glymphatic function may affect α-synuclein removal (Braun et al., 2024). A 2025 Brain study further indicated that glymphatic function influences α-synuclein propagation, supporting research into clearance pathways in synucleinopathies (Lopes et al., 2025).

Neuroimaging studies using DTI-ALPS and related MRI markers have reported imaging-derived, putative glymphatic dysfunction in Parkinson’s disease and isolated REM sleep behavior disorder, although these measures remain indirect and subject to vascular and white-matter confounding (Si et al., 2022). These findings do not show that lymphatic failure causes Parkinson’s disease. They support only the feasibility of evaluating clearance-related imaging phenotypes in prodromal and clinical synucleinopathy cohorts.

The MDS research criteria for prodromal Parkinson’s disease incorporate RBD, olfactory loss, subtle motor signs, autonomic dysfunction, and other markers in a probabilistic framework for identifying prodromal disease (Berg et al., 2015; Heinzel et al., 2019). iRBD should therefore not be treated only as a sleep disorder. It provides a biologically rich window into early α-synucleinopathy, future phenoconversion, and abnormal sleep architecture.

In the Parkinson’s Progression Markers Initiative cohort, CSF α-synuclein SAA detected biological heterogeneity among patients with PD and prodromal features such as hyposmia and RBD (Siderowf et al., 2023). In the DeNoPa cohort, α-synuclein seeds were detected in the CSF of patients with Parkinson’s disease and isolated RBD (Concha-Marambio et al., 2023). These assays can detect seeding-competent α-synuclein species when combined with longitudinal clinical, imaging, and sleep measures. They do not, however, directly measure propagation rate. A 2025 review also emphasizes exercise-related sleep improvement in synucleinopathies (Canonichesi et al., 2025); this supports the sleep pathway, not direct evidence of altered brain clearance.

The specific claim for PD is therefore this: in individuals with prodromal sleep phenotypes and biomarker evidence of synucleinopathy, timed exercise may matter most before widespread motor-stage disease. The premise is not that exercise directly dissolves Lewy bodies. Exercise may influence glymphatic function, autonomic rhythm, vascular pulsatility, α-synuclein processing, and sleep consolidation. If these mechanisms are relevant, timed exercise should produce measurable effects on sleep architecture, rest-activity rhythm stability, glymphatic imaging surrogates, α-synuclein SAA parameters, and phenoconversion trajectories.

This framework also implies that PD exercise trials should not be restricted to motor outcomes. MDS-UPDRS scores are clinically important, but they are heterogeneous and late endpoints for a clearance-based hypothesis. Prodromal or early Parkinson’s disease trials should include polysomnography, RBD severity, actigraphy-derived rest-activity rhythm metrics, autonomic markers, DAT imaging, α-synuclein SAA, and neurolymphatic imaging surrogates. These biomarkers are needed to distinguish symptomatic motor improvement from altered α-synuclein disease biology.

#### 3.3.4 Production, redistribution, or clearance? Mechanistic ambiguity

Mechanistic overinterpretation is a major risk in this field. Changes in tau or α-synuclein biomarkers do not automatically indicate enhanced clearance. Protein biomarkers reflect dynamic equilibria among production, release, phosphorylation, aggregation, degradation, extracellular transport, CSF exchange, blood transfer, and assay-specific detection. Exercise could affect any of these steps. A reduction in plasma p-tau217, for example, could reflect altered CSF-to-blood transport, reduced phosphorylation, reduced neuronal activity-dependent release, reduced downstream neurodegeneration, or altered peripheral clearance. Changes in α-synuclein SAA kinetics may reflect seed abundance, conformation, compartmental distribution, or assay amplification properties rather than reduced brain propagation.

This ambiguity is especially important for glymphatic hypotheses. Timed exercise could improve DTI-ALPS or meningeal lymphatic enhancement without improving tau or α-synuclein clearance. Conversely, plasma p-tau217 or α-synuclein SAA parameters could change without glymphatic mediation. Accurate inference requires convergence across multiple domains. A clearance-based mechanism would be strengthened if exercise timing produced a predictable temporal sequence of changes in sleep architecture, vascular pulsatility, neurolymphatic imaging markers, and protein biomarkers. It would be weakened if protein biomarkers changed without corresponding changes in sleep or clearance-related physiology.

The tau literature illustrates this problem clearly. Sleep loss increases ISF or CSF tau levels, but this may reflect changes in extracellular space, phosphorylation, clearance, neuronal activity-dependent release, or several processes at once (Holth et al., 2019; Barthélemy et al., 2020). Likewise, glymphatic inhibition can worsen tau aggregation and propagation in animal models (Harrison et al., 2020; Ishida et al., 2022; Lopes et al., 2024), but translating this finding to humans requires caution. Human tau PET measures aggregated fibrillar tau over months or years, whereas CSF and plasma p-tau reflect soluble protein dynamics over shorter timescales. These metrics are related but not interchangeable. Trials combining tau PET, plasma p-tau217, and sleep-glymphatic measurements will be more informative than trials relying on a single endpoint.

α-synuclein biomarkers are similarly ambiguous. Seed amplification assays detect seeding-competent α-synuclein but do not provide a quantitative measure of total brain α-synuclein burden or propagation rate. DAT imaging reflects dopaminergic terminal integrity rather than α-synuclein itself. RBD conversion reflects clinical phenoconversion and is influenced by comorbidities and heterogeneous disease pathways. Glymphatic imaging surrogates may reflect perivascular water diffusion, vascular integrity, or white-matter microstructure rather than α-synuclein clearance. Timed-exercise trials in prodromal Parkinson’s disease therefore need designs that can distinguish changes in sleep, clearance physiology, α-synuclein seeding activity, and neurodegenerative progression.

The framework should not be abandoned because of this ambiguity. Instead, ambiguity makes the hypothesis testable. Four predictions follow. First, matched-dose timing arms should produce different sleep, circadian, and vascular signatures. Second, sleep-compatible timing should have stronger effects on downstream protein endpoints than timing that disrupts sleep. Third, tau-related endpoints should be more responsive than amyloid burden in preclinical AD. Fourth, biomarker effects in prodromal Parkinson’s disease should be more detectable in individuals with RBD or α-synuclein SAA positivity than in unselected older adults. These predictions do not establish mediation: mediator-outcome confounding, treatment-induced confounding, regression to the mean, and measurement timing must be considered. Repeated mediator measurement and a prespecified longitudinal interventional-mediation analysis are required before a causal indirect effect is claimed.

Timed exercise should be viewed as an experimental perturbation of temporal physiology. Rather than merely increasing physical activity, the intervention should regulate the timing of vascular, autonomic, and metabolic stimulation relative to sleep and the circadian cycle. This design can determine whether exercise timing influences protein propagation, improves general health, or acts through unrelated pathways. A rigorous test should include accelerometry, polysomnography, circadian phase markers, cerebrovascular measures, neurolymphatic imaging, tau PET, plasma or CSF p-tau217, α-synuclein SAA, and longitudinal clinical outcomes.

Protein propagation provides disease-specific targets that generic neuroprotection does not. In AD, the most compelling target is tau spread in amyloid-positive individuals before widespread neocortical involvement. In Parkinson’s disease, the most compelling target is α-synuclein propagation in prodromal sleep phenotypes, especially iRBD and SAA-positive states. The glymphatic-meningeal lymphatic axis provides a plausible clearance interface, but its role should be tested rather than presumed. The purpose is not to claim that exercise has already been shown to alter proteinopathy. The goal is to define the biomarker architecture needed to determine whether timed exercise can affect early α-synuclein and tau spread.

### 3.4 Sleep, timing, and 24-h movement patterns as therapeutic variables

#### 3.4.1 Circadian entrainment and morning exercise

Morning exercise may be an effective strategy for stabilizing circadian organization in some older adults and those at risk of neurodegenerative disease. Exercise is a non-photic zeitgeber whose phase-shifting effects depend on circadian phase, intensity, duration, and chronotype (Van Reeth et al., 1994; Buxton et al., 1997; Lewis et al., 2018; Youngstedt et al., 2019). Morning exercise should therefore be tested against habitual wake time and circadian phase, not assumed to be beneficial merely because it occurs before noon. In a late chronotype, forcing an early session may increase sleep restriction, sympathetic activation, or circadian misalignment and could offset any entrainment benefit.

Morning exercise may improve the stability of the rest-activity rhythm, making it relevant to neurodegeneration. Weakened circadian amplitude, fragmented daily activity, altered sleep timing, and increased daytime inactivity are frequently associated with aging, Alzheimer’s disease, and Parkinson’s disease (Leng et al., 2019; Wang et al., 2026). These changes could be caused by autonomic dysfunction, reduced light exposure, low physical activity, neurodegenerative involvement of sleep-wake networks, medication side effects, or institutional routines. Morning exercise may counteract some of these processes by consolidating daytime activity, increasing exposure to outdoor light when done outside, moving activity timing forward, and reinforcing the behavioral contrast between active day and restful night-time physiology. Stronger daytime entrainment may improve subsequent sleep organization and thus support sleep-dependent neurolymphatic clearance, according to a temporal clearance model.

Exercise in the morning may thus serve two purposes: first, as a direct physical stimulus, and second, as a behavioral anchor that stabilizes rhythm. This review focuses on the second role. Exercise may have a greater influence on disease outcomes if it improves nightly physiological conditions for tau and α-synuclein handling, rather than simply increasing acute energy expenditure.

However, exercising in the morning is not universally appropriate. Some older adults may be unable to exercise safely because of orthostatic hypotension, joint stiffness, medication timing, decreased alertness, cold exposure, or fall risk. In Parkinson’s disease, morning “off” periods, akinesia, and autonomic instability may make afternoon exercise the primary safe and feasible window rather than merely an alternative vascular stimulus. Morning exercise should be used only when medication state, blood pressure, gait safety, sleep opportunity, and chronotype are compatible; otherwise, safety and adherence should take priority over theoretical entrainment benefits.

The most direct testable hypothesis is that people with delayed or fragmented rhythms will benefit more from morning exercise than those whose morning activity is already consistent. Future research should examine baseline activity timing, chronotype, sleep regularity, light exposure, and, if possible, dim-light melatonin onset. Changes in the amplitude of the rest-activity rhythm, sleep timing, slow-wave sleep, nocturnal autonomic tone, and clearance-related imaging markers, as well as step count and adherence, should be considered when evaluating a morning intervention. Morning exercise may have a behavioral rather than disease-modifying effect if it improves sleep architecture and moves the circadian phase without affecting neurolymphatic or protein biomarkers. The temporal intervention model is supported if it improves both tau and α-synuclein trajectories, as well as sleep-coupled clearance-related markers.

#### 3.4.2 Vascular-metabolic stimulation and afternoon exercise

Afternoon exercise may engage a different physiological pathway. Whereas morning exercise is best conceptualized as a circadian entrainment strategy, afternoon exercise may function primarily as a vascular-metabolic stimulus. Body temperature, muscle function, vascular responsiveness, and autonomic balance vary across the day and influence exercise performance. Some individuals may therefore tolerate higher intensity in the afternoon while remaining sufficiently distant from bedtime to avoid sleep disruption. This timing may be particularly suitable for older adults, patients with Parkinson’s disease who experience morning motor fluctuations, and individuals whose morning stiffness or hypotension limits safe activity.

The vascular rationale is central. Neurovascular coupling, cerebrovascular reactivity, and arterial pulsatility all influence lymphatic transport (Iliff et al., 2013; Mestre et al., 2018b; Holstein-Rønsbo et al., 2023). Long-term training improves arterial stiffness, endothelial function, and blood pressure regulation, whereas acute exercise increases heart rate, stroke volume, vascular shear stress, and cerebral perfusion demand. Afternoon exercise may provide a daily hemodynamic stimulus during the active phase without interfering with sleep onset. In the proposed model, this stimulus may help preserve vascular dynamics that support sleep-dependent clearance. This does not mean that afternoon exercise directly opens glymphatic channels. A more specific hypothesis is that appropriately timed vascular stimulation may preserve or improve the physiological drivers of nocturnal CSF-ISF exchange.

Cardiovascular research suggests that exercise responses may vary by time of day. Studies comparing morning and evening aerobic training in adults with hypertension have reported different effects on blood pressure, autonomic regulation, and baroreflex sensitivity (Brito et al., 2018, 2019, 2024). Systematic reviews of exercise timing and cardiovascular risk factors conclude that the evidence remains inconsistent, but that timing may influence selected vascular and autonomic outcomes (Sevilla-Lorente et al., 2023). These findings matter because vascular function is a plausible upstream regulator of neurolymphatic physiology, even though the studies themselves were not conducted in neurodegenerative populations. The timing most relevant for clearance-related endpoints may therefore differ from the timing most relevant for circadian entrainment.

Afternoon exercise may also be more practical in prodromal or early Parkinson’s disease. Patients with Parkinson’s disease often experience medication-related fatigue, autonomic dysfunction, sleep disturbance, and motor fluctuations. A strict morning regimen may be biologically plausible but poorly tolerated. Afternoon exercise may improve safety and adherence by allowing meals, wakefulness, and medication effects to stabilize. It may also reduce the risk that late, high-intensity exercise disrupts sleep. For these reasons, afternoon exercise is a strong candidate for matched-dose timing trials.

The main weakness of the afternoon vascular-metabolic model is that the evidence remains indirect. No study has shown that afternoon exercise improves glymphatic or meningeal lymphatic clearance more than morning or evening exercise. Accordingly, afternoon exercise should be framed as a testable hypothesis and, for participants with morning “off” periods or orthostatic risk, a safety-prioritized primary window. The relevant experiment would compare morning and afternoon exercise under equivalent prescribed dose while monitoring completed workload, internal load, sleep, vascular pulsatility, cerebrovascular reactivity, imaging-derived neurolymphatic surrogates, and protein biomarkers.

A further nuance is that afternoon exercise may interact with sedentary accumulation earlier in the day. If a person sits for 6 or 7 h before exercising, a single afternoon session may not fully reverse prolonged reductions in vascular stimulation. Afternoon exercise may therefore need to be combined with sedentary breaks rather than treated as a stand-alone intervention. Neurodegenerative prevention may require more than scheduling one exercise session; it may require shaping the entire daily movement pattern so that vascular, metabolic, and sleep-related signals remain coherent across the 24-h cycle.

#### 3.4.3 Evening exercise and sleep-gated clearance

Evening exercise is the most clinically ambiguous timing strategy. Older sleep-hygiene advice often discouraged exercise near bedtime, whereas more recent systematic reviews indicate that appropriately timed, moderate-intensity evening exercise does not necessarily impair sleep and may improve some sleep outcomes (Stutz et al., 2019; Frimpong et al., 2021; Yue et al., 2022; Kim et al., 2023). The discrepancy depends on intensity, duration, fitness, chronotype, baseline sleep quality, and the interval between exercise and bedtime. For a neurolymphatic framework, the relevant question is not whether evening exercise is generally beneficial or harmful. The relevant question is whether it supports or disrupts the subsequent sleep architecture through which clearance is likely to be gated.

Meta-analyses in healthy adults suggest that evening exercise performed sufficiently before bedtime usually does not impair sleep, although very late or high-intensity exercise may be less favorable (Stutz et al., 2019; Frimpong et al., 2021; Yue et al., 2022). A large free-living study using wearable data reported a dose-response pattern in which later timing and higher exercise strain were associated with delayed sleep onset, shorter sleep duration, lower sleep quality, higher nocturnal resting heart rate, and lower heart rate variability (Leota et al., 2025). Exercise ending at least 4 h before sleep onset was not associated with these adverse sleep outcomes. This finding shifts the issue from evening exercise *per se* to exercise strain relative to sleep onset.

This distinction is critical for Alzheimer’s and Parkinson’s disease. In this framework, sleep is not simply an outcome of exercise; it may be a clearance gate. CSF-ISF exchange and metabolite clearance have been linked to slow-wave sleep and low-arousal states (Xie et al., 2013; Holth et al., 2019; Barthélemy et al., 2020). Evening exercise may support nocturnal clearance if it improves sleep continuity, reduces stress, improves glucose regulation, or promotes relaxation. By contrast, high-strain exercise close to bedtime may undermine a pathway relevant to tau and α-synuclein handling by increasing sympathetic activity, delaying sleep onset, or reducing restorative sleep.

Vigorous evening exercise and low-intensity evening movement should therefore be evaluated separately. After dinner, low-intensity cycling, stretching, or light walking may improve postprandial glucose handling, interrupt prolonged sitting, and prepare the body for sleep without excessive autonomic load. In vulnerable individuals, however, prolonged vigorous exercise or high-intensity interval training close to bedtime may increase catecholamines, nocturnal heart rate, and core temperature. Patients with insomnia, RBD, Parkinson’s disease-related autonomic dysfunction, and older adults may be particularly sensitive to these effects.

A major limitation of current evening-exercise studies is that most do not assess neurodegenerative endpoints. They typically measure heart rate, sleep duration, sleep efficiency, sleep-onset latency, and subjective sleep quality, but rarely quantify slow-wave sleep, CSF dynamics, imaging-derived neurolymphatic markers, or protein biomarkers. Evidence from systematic reviews in healthy adults and older adults, and from Parkinson’s disease sleep trials, supports feasibility but does not establish a disease-specific clearance effect (Stutz et al., 2019; Amara et al., 2020; Cristini et al., 2021; Frimpong et al., 2021; Fank et al., 2022; Yue et al., 2022; Kim et al., 2023; Páez et al., 2024; Geng et al., 2025). Evening exercise should therefore be classified by strain and time to sleep onset, not treated as uniformly beneficial or harmful.

Trial designs should not exclude evening exercise; they should classify it by timing and intensity relative to sleep onset. A reasonable design would avoid high-strain exercise within a prespecified pre-bedtime window and include an evening low-intensity arm, especially in individuals with poor sleep quality or autonomic dysfunction. Outcomes should include polysomnography, slow-wave sleep, REM sleep characteristics, nocturnal heart-rate variability, subjective sleep quality, next-day alertness, and clearance-related imaging. In prodromal PD or RBD, REM sleep architecture and REM sleep without atonia require particular attention.

The logical conclusion is conditional: evening exercise may be beneficial if it improves the physiology of sleep-gated clearance, but detrimental if it disrupts the sleep architecture required for that clearance. This conditionality is not a weakness. It is precisely why exercise timing should be tested experimentally rather than assumed.

#### 3.4.4 Daytime vascular dynamics and sedentary fragmentation

The temporal model should not focus only on exercise sessions. The distribution of sedentary time across the day may also be biologically relevant. Prolonged sitting can alter cerebral hemodynamics, reduce vascular shear stress, lower metabolic demand, and decrease skeletal-muscle activity. Because AD and PD pathology can interact with cerebral hypoperfusion, vascular stiffness, and impaired cerebrovascular reactivity, these effects are relevant to neurodegeneration. If vascular pulsatility and neurovascular coupling influence glymphatic function, prolonged daytime inactivity may create a low-stimulation vascular environment that is poorly captured by daily step counts or weekly exercise minutes.

Experimental evidence supports this concern. In healthy desk workers, prolonged uninterrupted sitting reduced middle cerebral artery blood-flow velocity, whereas frequent brief walking breaks reversed this trend (Carter et al., 2018). Morning exercise in older adults also reduced the adverse influence of prolonged sitting on cerebral blood flow (Wheeler et al., 2019), supporting the idea that activity distribution can influence cerebrovascular dynamics throughout the day. Other studies indicate that prolonged sitting can alter cerebrovascular hemodynamics, whereas reducing sedentary behavior or increasing activity breaks may improve cerebral blood flow and vascular health (Maasakkers et al., 2020; Taylor et al., 2022; Horiuchi et al., 2023; Tallon et al., 2023). These findings provide a physiological basis for treating sedentary fragmentation as a neurovascular factor rather than a behavioral nuisance.

This evidence is especially relevant for older adults and for populations with prodromal neurodegenerative disease. Many individuals at risk for Alzheimer’s disease or Parkinson’s disease may be unable or unwilling to undertake sustained moderate-to-vigorous exercise. Sedentary-break interventions may therefore be more feasible than structured training. A prescription such as 3–5 min of light walking every 30–60 min could repeatedly stimulate skeletal muscle, blood flow, autonomic regulation, and glucose handling, even if it produces little change in cardiorespiratory fitness. Within a clearance framework, the key question is whether this distributed movement pattern supports vascular conditions that aid nocturnal clearance or reduces daytime physiological states that inhibit it.

Sedentary fragmentation may also influence sleep. Prolonged daytime inactivity can reduce behavioral contrast between day and night, increase daytime napping, and weaken sleep pressure. Frequent light activity may increase daytime arousal and improve sleep consolidation, whereas excessive evening sedentary behavior followed by late vigorous exercise may produce a mismatched pattern: insufficient movement during the day and excessive strain near bedtime. The timing of sedentary breaks may therefore matter. Morning and early-afternoon breaks may support daytime vascular dynamics and circadian amplitude; late-evening breaks are likely beneficial only if they are low intensity and do not interfere with sleep.

This perspective changes how accelerometry data should be interpreted. Total sedentary time is informative but incomplete. Trials should report sedentary-bout duration, break frequency, timing of the longest bouts, transitions between sedentary and active states, and, as an exploratory temporal-phenotyping measure, the fitted power-law exponent (alpha) of the sedentary-bout distribution. The exponent must be defined with a prespecified wear-time, bout-detection, minimum-bout, and fitting procedure because it is not yet a validated proteinopathy endpoint. Twenty-four-hour behavior should not be reduced to daily step counts.

Sedentary fragmentation may be particularly relevant in amyloid-positive individuals without cognitive impairment, because subtle behavioral changes may precede overt decline. In prodromal and early-stage Parkinson’s disease, fatigue, apathy, constipation, orthostatic symptoms, and sleep problems can all reduce spontaneous movement. Sedentary-break interventions could provide a low-risk platform for testing sleep-mediated and vascular mechanisms in both conditions. They could also be combined with morning or afternoon exercise, allowing researchers to distinguish the effects of a single exercise session from the effects of distributed movement across the day.

However, proteinopathy endpoints have not yet been tested in sedentary-break interventions. Current evidence suggests that cerebral blood flow and vascular function are more likely to change than tau, α-synuclein, or glymphatic clearance. This distinction should be explicit. Sedentary fragmentation may be a therapeutic variable because it alters plausible upstream physiology, but biomarker evidence is still required before claiming disease-modifying potential.

#### 3.4.5 Timing based on chronotype and disease stage

The main implication of the temporal framework is that there may be no single best time to exercise. Chronotype, sleep phenotype, vascular status, medication schedule, disease stage, and safety constraints may modify the effect of timing. Baseline chronotype, disease stage, vascular risk, habitual sleep, and baseline activity should be treated as pre-exposure covariates and candidate effect modifiers; post-assignment sleep, circadian, autonomic, and vascular changes are candidate mediators. A timing prescription that is mismatched to chronotype may worsen sleep and should be treated as a possible harm, not merely as non-adherence.

A 2026 randomized trial in middle-aged adults at high cardiovascular risk found that matching exercise timing to individual chronotype improved cardiometabolic and sleep-related outcomes more than mismatched timing (Tariq et al., 2026). Although that study was not conducted in neurodegenerative disease, it supports measuring chronotype and phase angle rather than assigning broad clock-time categories. Neurodegenerative trials should assess actigraphy-derived sleep timing, validated questionnaires, light exposure, medication timing, and, where feasible, dim-light melatonin onset. Chronotype matching remains an effect-modification hypothesis, not proof that a particular clock time improves protein handling.

Disease stage is equally important. In preclinical Alzheimer’s disease, amyloid-positive but tau-negative or tau-limited individuals may be the most appropriate candidates if the goal is to slow tau spread rather than reverse established dementia. This population may benefit from morning exercise for rhythm stabilization, afternoon exercise for vascular stimulation, or evening low-intensity activity for sleep preparation, depending on baseline sleep and activity phenotype. Morning entrainment may be most relevant for an amyloid-positive individual with delayed sleep timing and low morning activity; afternoon exercise plus sedentary breaks may be more relevant for someone with vascular risk and prolonged daytime sitting; and late high-strain exercise should be avoided in individuals with insomnia.

The timing problem is different in prodromal Parkinson’s disease. Individuals with iRBD or α-synuclein SAA positivity often show sleep disruption before formal motor diagnosis. Avoiding interventions that worsen REM sleep instability, nocturnal autonomic dysfunction, orthostatic symptoms, or falls is the first priority. Morning or afternoon exercise may therefore be safer as primary intervention arms; in participants with morning “off” periods or hypotension, afternoon should be prespecified as the primary safety-prioritized window, whereas evening exercise should remain low intensity and closely monitored.

Sex, age, and comorbidity also need to be considered when matching timing to chronotype and disease stage. Exercise tolerance, vascular responses, and sleep architecture may differ between older men and women. Hypertension, diabetes, sleep apnea, depression, frailty, and polypharmacy can all influence both exercise response and neurodegenerative risk. Meals, light exposure, and medication regimens also shape when exercise is physiologically feasible. A high-level framework should therefore avoid simplistic claims such as “morning exercise is best” or “evening exercise is harmful.” The stronger claim is that exercise timing should match the intended mechanism and the individual’s physiological constraints.

This approach provides a classification strategy for future experiments. Before randomization, participants could be categorized by rhythm phenotype: delayed or fragmented rhythm, low-amplitude rhythm, excessive daytime sedentary accumulation, insomnia, RBD, vascular risk, and frailty or autonomic risk. Exercise timing could then be aligned with the proposed mechanism: morning entrainment for delayed rhythms, afternoon vascular-metabolic stimulation for sedentary or vascular-risk phenotypes, evening low-intensity movement for sleep preparation and postprandial sedentary accumulation, and distributed sedentary breaks for individuals with low exercise tolerance. Such a design is more complex than a standard exercise trial, but it tests timing as a mechanistic variable rather than as an adherence preference.

Falsifiability is essential. If chronotype-matched exercise improves vascular and sleep outcomes but not neurolymphatic or protein biomarkers, timing may be clinically useful without being disease-modifying. If timing improves tau or α-synuclein endpoints only in a biomarker-defined subgroup, it should be treated as a precision-medicine strategy rather than as general public-health advice. If total dose explains outcomes better than timing after strict control for sleep, circadian phase, and disease stage, the dose model remains primary. The field needs experiments that can produce any of these outcomes.

Timing, sleep, and 24-h movement behavior should therefore be treated as therapeutic variables because they determine how exercise is integrated into daily physiology. Sedentary breaks may preserve daytime vascular dynamics; morning exercise may entrain rhythms; afternoon exercise may stimulate vascular and metabolic systems; evening exercise may either support or impair sleep-gated clearance; and prescriptions tailored to chronotype or disease stage may determine whether these strategies are safe, adherent, and biologically relevant. The next step is to test these timing hypotheses with clearance and protein-propagation endpoints, rather than relying only on activity volume or subjective sleep quality.

### 3.5 Biomarker-driven trials of temporal neurotherapeutics

#### 3.5.1 Intervention windows: prodromal Parkinson’s disease and preclinical Alzheimer’s disease

Disease-stage specificity is required for the temporal intervention framework. Exercise timing is unlikely to have the same biological relevance across the neurodegenerative continuum. In late-stage AD or advanced PD, widespread protein pathology, neuronal loss, sleep disruption, vascular dysfunction, and reduced mobility may substantially limit the capacity of exercise to alter disease biology. By contrast, pathogenic protein processes can be detected in preclinical AD and prodromal PD before clinical impairment and structural degeneration are fully established, making these stages more appropriate testing grounds.

The amyloid-positive, tau-negative, or tau-limited stage of Alzheimer’s disease offers the clearest intervention window. The NIA-AA preclinical AD framework allows Alzheimer’s disease to be staged biologically before symptoms emerge using amyloid, neurodegeneration, and downstream biomarker evidence (Sperling et al., 2011). More recent biological criteria have reinforced the use of core biomarkers, rather than clinical syndromes alone, to define and stage Alzheimer’s disease (Jack et al., 2024). This shift directly affects exercise trials. If the proposed target is tau propagation rather than generic cognitive reserve, participants should be enriched by amyloid and tau biomarkers rather than selected only by age, subjective memory complaint, or family history.

Longitudinal imaging studies support this staging logic. Combined tau and amyloid positivity is associated with subsequent cognitive decline in cognitively unimpaired older adults, and tau measures correlate more strongly with short-term clinical progression than amyloid alone (Hanseeuw et al., 2019; Sperling et al., 2024). Baseline tau and amyloid levels predicted cognitive and functional decline in the A4 and LEARN cohorts, supporting biomarker enrichment in secondary-prevention trials (Sperling et al., 2024). Tau PET analyses further show that tau accumulation in preclinical AD is subtle and regionally heterogeneous, requiring careful population selection and longitudinal follow-up (Insel et al., 2023). If a timed-exercise trial aims to test disease modification, large unselected samples of older adults are a poor choice. The appropriate population is one in which tau accumulation is measurable, biologically plausible, and not yet anatomically saturated.

Blood biomarkers could make this enrichment strategy more feasible. Plasma p-tau217 can detect longitudinal change, including in preclinical stages, and shows high diagnostic accuracy for biological AD (Ashton et al., 2024; Rissman et al., 2024). These markers can reduce screening burden, permit repeated sampling, and enable temporal coupling between exercise exposure, sleep physiology, and protein dynamics. They cannot, however, fully replace PET or CSF in mechanistic trials. Plasma p-tau217 may serve as both an enrichment tool and a repeat intermediate endpoint, whereas tau PET provides a slower but more spatially specific measure of propagation.

The emergence of disease-modifying anti-amyloid therapies further supports early exercise trials. In early symptomatic AD, lecanemab and donanemab slowed clinical decline and produced biomarker effects, but both require early disease detection, safety monitoring, and biomarker-defined populations (Sims et al., 2023; van Dyck et al., 2023). Behavioral interventions should now be held to a similar biomarker-based standard. Timed exercise does not need to compete directly with monoclonal antibodies. Rather, it should be evaluated in the same biological disease space: early AD, biomarker-confirmed pathology, and longitudinal protein endpoints.

Prodromal synucleinopathy is the most promising window for Parkinson’s disease intervention. The MDS prodromal PD criteria provide a probabilistic framework that incorporates clinical and biomarker features such as RBD, olfactory dysfunction, subtle motor signs, autonomic symptoms, and dopaminergic imaging (Berg et al., 2015; Heinzel et al., 2019). The Neuronal alpha-Synuclein Disease Integrated Staging System and other recent biological staging frameworks propose staging individuals by α-synuclein biology and functional impairment across the disease continuum (Dam et al., 2024). These frameworks shift PD exercise trials away from late motor outcomes and toward biologically enriched populations.

Isolated RBD is particularly useful for timed-exercise trials. It directly involves sleep systems that are central to the present model and is strongly associated with future Parkinson’s disease, dementia with Lewy bodies, and multiple system atrophy (Postuma et al., 2015; Postuma et al., 2019). α-synuclein seed amplification assays provide a molecular biomarker for detecting synucleinopathy in prodromal and early-stage populations (Concha-Marambio et al., 2023; Siderowf et al., 2023; Bernhardt et al., 2025; Coughlin et al., 2025). In the PPMI cohort, individuals with clinically diagnosed Parkinson’s disease and at-risk groups, including those with RBD or hyposmia, showed α-synuclein SAA positivity (Siderowf et al., 2023). Recent studies suggest that SAA positivity and kinetic parameters may help predict risk in at-risk individuals (Bernhardt et al., 2025; Coughlin et al., 2025). These assays can enrich timed-exercise trials for underlying α-synuclein biology, although they do not yet provide a linear measure of propagation.

Skin biopsy for phosphorylated α-synuclein may also be useful for enrichment or stratification. A 2024 cross-sectional study reported high diagnostic performance for detecting phosphorylated α-synuclein in skin biopsies across clinically defined synucleinopathies (Doppler et al., 2017, 2021; Gibbons et al., 2024). Skin biopsy can detect peripheral synucleinopathy and autonomic involvement, but it does not directly measure brain α-synuclein propagation. This distinction matters in a timed-exercise framework because autonomic dysfunction may influence both neurolymphatic physiology and exercise safety.

Temporal neurotherapeutic trials should therefore target specific populations. For Alzheimer’s disease, priority should go to cognitively intact or very early MCI individuals with amyloid positivity and minimal or absent tau spread. For Parkinson’s disease, priority should go to individuals with iRBD and α-synuclein SAA positivity, hyposmic individuals with SAA positivity, and very early untreated PD. These groups are biologically appropriate but harder to recruit than broad clinical samples. A trial that enrolls participants after the propagation window has largely closed may incorrectly conclude that timing is irrelevant.

#### 3.5.2 Multimodal biomarkers for timed-exercise trials

Timed-exercise trials require multimodal biomarkers because no single measure can establish the proposed pathway. The central hypothesis is that exercise timing influences sleep, circadian alignment, vascular pulsatility, glymphatic-meningeal lymphatic clearance, and protein propagation. A credible trial must therefore measure each step of this pathway. Otherwise, changes in cognition or motor function cannot be interpreted mechanistically.

Wrist, hip, or thigh accelerometers should measure step count, moderate-to-vigorous physical activity, light-intensity activity, sedentary-bout duration, sedentary-break frequency, activity fragmentation, peak activity timing, and exercise timing relative to habitual wake time and sleep onset. Trials should distinguish prescribed external dose (minutes, target intensity, frequency), completed workload (pace, power, distance, duration), internal physiological load (heart rate, perceived exertion, heart-rate variability, and, where feasible, oxygen uptake), adherence, and non-intervention activity. These quantities should not be collapsed into one “dose” variable (Rosenberger et al., 2016; Ross et al., 2020).

Sleep and circadian physiology form the second domain. Actigraphy can measure sleep timing, sleep efficiency, and rest-activity rhythm stability, but it cannot define sleep architecture precisely. Mechanistic substudies should therefore include polysomnography, especially when the trial hypothesis involves RBD, REM sleep, or slow-wave sleep. Key endpoints include slow-wave sleep duration, sleep fragmentation, arousal index, REM sleep without atonia, sleep-onset latency, wake after sleep onset, and nocturnal autonomic indices. Circadian measures should include chronotype, light exposure, and, where feasible, dim-light melatonin onset. Rest-activity rhythm metrics such as amplitude, acrophase, intradaily variability, and interdaily stability should be prespecified rather than added *post hoc*. Traditional actigraphy studies in dementia and movement disorders have already shown both the utility and limitations of activity-based rhythm monitoring (van Someren et al., 1996).

The third domain is vascular and neurolymphatic physiology. Because human glymphatic and meningeal lymphatic imaging remains indirect, trials should not rely on a single marker. A reasonable panel would include DTI-ALPS, perivascular-space burden, arterial pulsatility indices, cerebrovascular reactivity, CSF-flow-sensitive MRI, and contrast-enhanced meningeal lymphatic imaging (Eide and Ringstad, 2015; Taoka et al., 2017; Zhou et al., 2020; Patel et al., 2023; Ringstad and Eide, 2024; Satpathi et al., 2025). These are imaging-derived, putative surrogates and should not be described as direct indicators of glymphatic flux or protein removal. A single positive DTI-ALPS or meningeal-enhancement change is insufficient to establish a clearance mechanism.

The fourth domain is disease-specific protein pathology. For AD, amyloid PET and tau PET remain the most spatially informative tools, whereas plasma p-tau217 is a repeatable soluble biomarker useful for enrichment and intermediate dynamics (Ashton et al., 2024; Rissman et al., 2024; Rabinovici et al., 2025). Tau PET and plasma p-tau217 are not interchangeable and neither directly measures clearance or propagation rate. For PD, α-synuclein SAA is disease-specific for underlying synucleinopathy, but positivity is not a linear measure of brain burden or propagation; DAT imaging, skin phosphorylated α-synuclein, and phenoconversion are complementary but distinct outcomes (Doppler et al., 2017, 2021; Concha-Marambio et al., 2023; Siderowf et al., 2023; Dam et al., 2024; Gibbons et al., 2024; Bernhardt et al., 2025; Coughlin et al., 2025). Operationally, “protein propagation” is treated here as a longitudinal outcome construct rather than as a single biomarker. In AD, the closest *in vivo* operational measure is longitudinal regional expansion and accumulation of tau-PET signal along prespecified anatomical or connectivity-informed trajectories; plasma or CSF p-tau217 is treated as a soluble disease-dynamics marker rather than a spatial propagation measure. In PD, no validated *in vivo* biomarker currently quantifies α-synuclein propagation rate. α-Synuclein SAA positivity or kinetics, skin phosphorylated α-synuclein, DAT or neuromelanin imaging, and phenoconversion are therefore complementary disease-process endpoints, not interchangeable measures of propagation. A propagation claim should require longitudinal change consistent with spread across regions or compartments rather than a change in a single biomarker.

For Parkinson’s disease, α-synuclein SAA is currently the most disease-specific molecular tool for *in vivo* synucleinopathy detection (Concha-Marambio et al., 2023; Siderowf et al., 2023; Bernhardt et al., 2025; Coughlin et al., 2025). Because SAA positivity alone does not define disease stage, propagation rate, or regional burden, it should be combined with complementary markers. Autonomic testing can detect peripheral involvement, DAT imaging assesses dopaminergic terminal integrity, neuromelanin MRI provides nigral information, and skin biopsy for phosphorylated α-synuclein detects peripheral synucleinopathy (Doppler et al., 2017, 2021; Gibbons et al., 2024). In iRBD trials, polysomnography is essential for diagnosis, stratification, and mechanistic interpretation.

The fifth domain is clinical outcome. Clinical endpoints should be treated as downstream outcomes, not as primary mechanistic evidence. A timed-exercise intervention may improve motor function or sleep without altering proteinopathy, and a biomarker effect may occur before measurable clinical benefit. Each trial should state one primary endpoint and a prespecified hierarchy of secondary domains; confirmatory analyses should be separated from exploratory biomarker panels, with a formal multiplicity strategy such as hierarchical testing or false-discovery-rate control.

The minimum dataset for a timed-exercise neurotherapeutic trial should include objectively measured exercise timing, 24-h movement composition, actigraphy, polysomnography in a mechanistic subset, sleep and circadian phase markers, vascular physiology, neurolymphatic imaging surrogates, disease-specific protein biomarkers, and safety/adherence outcomes. This is demanding, but a less complete dataset would be unable to distinguish timing effects from vascular fitness, sleep enhancement, general activity, or reverse causality.

#### 3.5.3 Exercise, timed walking, and sedentary breaks in prodromal Parkinson’s disease and preclinical Alzheimer’s disease

##### 3.5.3.1 Preclinical AD and timed walking

For preclinical AD, a timed-walking trial is the most direct initial design. The target population would be adults aged 60–80 years who are cognitively normal or have very early MCI, are amyloid-positive, and have negative or spatially restricted tau PET. Baseline sedentary or low-activity status should be required because highly active individuals may have limited capacity for behavioral change. Plasma p-tau217 could be used for initial screening, followed by amyloid PET and tau PET confirmation in the mechanistic cohort.

The intervention should compare timing under matched prescribed-dose conditions. In a straightforward design, participants would be randomized to morning walking, afternoon walking, or evening low-intensity walking; sedentary-break interventions should be tested as a separate design rather than pooled with session timing. The primary estimand should be the intention-to-treat effect of assigned timing on one prespecified primary endpoint. Timing, not total dose, is the experimental contrast; completed workload, internal load, adherence, light exposure, meals, medication timing, and non-intervention activity should be verified and reported.

Primary mechanistic endpoints should be selected one at a time. A short proof-of-mechanism study should designate one proximal endpoint, such as change in polysomnographic slow-wave sleep in preclinical AD or REM sleep without atonia in iRBD, with vascular and imaging-derived neurolymphatic measures as ordered secondary outcomes. A longer disease-modification study may instead prespecify one disease-specific endpoint, such as the slope of plasma p-tau217 or longitudinal regional tau-PET change in AD, or a clinical phenoconversion endpoint in prodromal PD. SAA positivity, SAA kinetics, DAT imaging, and clinical outcomes are not interchangeable and should be analyzed in a prespecified hierarchy.

The EXERT trial is instructive. It compared aerobic exercise with stretching, balance, and range-of-motion exercise and assessed cognitive and Alzheimer’s disease biomarker outcomes in sedentary older adults with amnestic MCI (Baker et al., 2025). The findings are important because they show that structured exercise trials with biomarker outcomes are feasible in cognitively impaired populations. However, EXERT was not designed to test tau propagation, sleep-gated clearance, or exercise timing as primary mechanisms. A temporal trial should go further by prospectively integrating sleep, clearance, and protein biomarkers while controlling dose across timing arms.

##### 3.5.3.2 Timed exercise in iRBD and prodromal Parkinson’s disease

The second priority is a timed-exercise trial in prodromal Parkinson’s disease, specifically PSG-confirmed iRBD with α-synuclein SAA positivity. This population is mechanistically aligned with the sleep-clearance hypothesis, clinically meaningful, and biologically heterogeneous. Participants should be stratified by RBD severity, autonomic dysfunction, chronotype, baseline activity rhythm, and SAA status. Individuals with severe orthostatic hypotension, unstable sleep disorders, or high fall risk should follow separate safety protocols.

A feasible design would compare morning circadian exercise, afternoon aerobic or resistance exercise, evening low-intensity movement and stretching, and a health-education control only as a secondary comparator. The primary comparison should be between the matched-dose exercise arms. In PSG-confirmed iRBD with α-synuclein SAA positivity, afternoon exercise should be prespecified as the safety-prioritized primary window for participants with morning “off” periods, orthostatic hypotension, or severe morning akinesia; high-intensity late-night exercise should initially be avoided.

Key mechanistic endpoints should include polysomnographic REM sleep characteristics, REM sleep without atonia, sleep fragmentation, actigraphy-derived rhythm stability, autonomic measures, α-synuclein SAA status or kinetic parameters, DAT imaging, and neurolymphatic imaging surrogates. Clinical outcomes should include RBD severity, olfaction, constipation or autonomic symptoms, MDS-UPDRS, and longitudinal phenoconversion. If the trial is short, it should be presented as a mechanistic study rather than as a definitive disease-modification trial.

Previous PD exercise trials provide guidance on safety and feasibility. The SPARX phase 2 trial found that high-intensity treadmill exercise was safe and feasible in *de novo* Parkinson’s disease (Schenkman et al., 2018). The Park-in-Shape trial and its neuroimaging follow-up studies showed that remotely supervised, gaming-supported, home-based aerobic exercise improved motor outcomes (van der Kolk et al., 2019; Johansson et al., 2022). These trials are important, but they primarily address brain structure and function, motor outcomes, intensity, and feasibility. They do not test whether exercise timing affects sleep-dependent α-synuclein biology. Future trials should preserve their practical strengths while adding timing, sleep, and α-synuclein biomarker endpoints.

##### 3.5.3.3 Sedentary-break interventions

The third design is a sedentary-break trial. This tests a distinct temporal hypothesis, not merely a less intensive version of exercise training. If prolonged sitting compromises cerebral hemodynamics and vascular stimulation, breaking up sedentary time may improve daytime vascular dynamics and indirectly support sleep-gated clearance. This design may be especially useful for participants who cannot tolerate prolonged moderate-intensity exercise, including older adults, individuals with preclinical AD or MCI, people with iRBD, and patients with early Parkinson’s disease.

The intervention could prescribe 3–5 min of light walking or sit-to-stand activity every 30–60 min during waking sedentary periods. The control group could receive a matched attention intervention or general activity advice. Expected outcomes include sedentary-bout duration, break frequency, cerebral blood-flow velocity, cerebrovascular reactivity, postprandial glucose, sleep efficiency, rest-activity rhythm amplitude, glymphatic imaging surrogates, and protein biomarkers in enriched subgroups. Acute crossover studies could first test vascular and sleep effects before longer trials evaluate protein endpoints.

The vascular plausibility of this approach is supported by prolonged-sitting studies. Prolonged sitting can reduce cerebral blood-flow velocity, and walking breaks or morning exercise can offset some of these effects (Carter et al., 2018; Wheeler et al., 2019). More recent crossover studies are examining whether interrupting prolonged sitting improves cognition and cerebral blood flow in older adults (Cunha et al., 2025). These studies do not demonstrate disease modification. A sedentary-break trial in neurodegeneration should therefore be framed as a test of an upstream physiological mechanism, not as an established anti-propagation intervention.

##### 3.5.3.4 Core design principles

The three trial types should share several core principles. First, timing arms must be matched for prescribed external dose, while completed workload, internal physiological load, adherence, and non-intervention activity are analyzed separately. Second, baseline rhythm phenotype and chronotype should be assessed before randomization. Third, sleep should be treated as both an outcome and a candidate mediator, not merely as a confounder. Fourth, one primary estimand and one primary endpoint should be defined for each trial, followed by a prespecified hierarchy of secondary outcomes. Fifth, confirmatory and exploratory analyses should be separated and multiplicity controlled. Sixth, sequential biomarker changes do not prove mediation; repeated mediator measurement, mediator-outcome confounding assessment, and a prespecified longitudinal interventional-mediation analysis are required.

#### 3.5.4 Limitations, controversies, and falsifiable predictions

Several limitations must be made explicit. First, neurolymphatic imaging in humans remains indirect: DTI-ALPS, perivascular-space burden, contrast-enhanced meningeal lymphatic imaging, and CSF-flow measures are imaging-derived surrogates and do not directly measure tau or α-synuclein clearance. Second, exercise timing is difficult to disentangle from light exposure, meals, medication schedules, social rhythms, sleep opportunity, internal load, and adherence. Third, chronotype mismatch may worsen sleep and should be analyzed as a possible harm or effect modifier. Fourth, protein biomarkers may change for reasons unrelated to clearance. Fifth, long-term biomarker trials are expensive, time-consuming, and vulnerable to declining adherence. The primary exercise-group contrast, one primary endpoint, and multiplicity plan should be specified before enrollment.

Alzheimer’s disease biomarkers require particular caution. Plasma p-tau217 is promising, but changes during an exercise trial could reflect altered phosphorylation, neuronal activity, clearance, blood-brain barrier transport, or peripheral kinetics. Tau PET is more spatially specific but evolves slowly and requires long follow-up. Amyloid PET may remain unchanged even if tau-related processes change. Therefore, a trial designed around tau propagation should not be judged negative simply because amyloid burden does not change.

PD biomarkers are similarly difficult to interpret. α-synuclein SAA is a major advance, but subtle intervention effects may not be captured by a binary positive/negative result. Quantitative kinetic measures may provide additional information, although their longitudinal meaning is still being defined (Bernhardt et al., 2025; Coughlin et al., 2025). DAT imaging measures dopaminergic terminal integrity, not α-synuclein burden. Skin biopsy reflects peripheral synuclein deposition rather than brain propagation. RBD phenoconversion is clinically meaningful but slow and variable. A biomarker-driven PD trial should therefore combine molecular, imaging, sleep, and clinical outcomes rather than rely on a single readout.

Safety is not secondary. Exercise timing can affect orthostatic symptoms, fall risk, medication interactions, fatigue, and sleep in older adults and patients with Parkinson’s disease. Morning exercise may be unsafe for individuals with severe morning akinesia or orthostatic hypotension. Evening exercise that is too intense or too close to bedtime may be problematic for individuals with insomnia, RBD, or autonomic instability. Sedentary breaks may be safer but may produce smaller physiological effects. Trial outcomes should include adverse sleep effects, falls, cardiovascular events, musculoskeletal injuries, and adherence.

The framework is testable despite these uncertainties. Under matched prescribed-dose conditions, timing should matter if morning, afternoon, and evening exercise produce distinct signatures in rest-activity rhythm, sleep architecture, nocturnal autonomic tone, and vascular physiology. Sleep-compatible timing should produce stronger imaging-derived surrogate effects than timing that disrupts sleep, but a surrogate change without a corresponding protein or clinical change should not be interpreted as disease modification. In preclinical AD, tau-related endpoints should be prioritized over amyloid burden if the proposed downstream pathway is correct. In prodromal Parkinson’s disease, effects should be more detectable in α-synuclein SAA-positive or PSG-confirmed iRBD groups. Clearance mediation should be inferred only from convergent longitudinal evidence, not from a single biomarker or from sequential observations alone. Confirmatory analyses should use a prespecified primary endpoint and multiplicity control; the remaining biomarker and subgroup analyses should be labelled exploratory.

Biomarker-driven trials are the critical next step. The central question is not whether physical activity is generally healthy. It is whether exercise can be prescribed as a temporally gated intervention that affects sleep and vascular physiology, changes imaging-derived neurolymphatic surrogates, and alters disease-specific protein dynamics while tau and α-synuclein propagation remain modifiable. Answering that question will require narrowed populations, objective phase-aware timing measures, a primary exercise-group estimand, multimodal but hierarchically ordered biomarkers, and prespecified mediation and multiplicity analyses.

## 4 Conclusion

Exercise is among the most accessible and biologically broad interventions for older adults at risk of neurodegenerative disease. Its current conceptualization, however, remains incomplete. Most studies continue to define exercise by dose, intensity, duration, or fitness gain, even though neurodegenerative proteinopathies develop within a temporally structured physiological environment. Sleep, circadian rhythms, vascular pulsatility, autonomic regulation, and 24-h movement organization may shape the conditions under which misfolded proteins are released, redistributed, degraded, or propagated.

The glymphatic-meningeal lymphatic axis offers a plausible interface between timed exercise and proteinopathy biology. Animal studies show that exercise, sleep state, vascular dynamics, and AQP4-dependent mechanisms can influence lymphatic transport, whereas human studies provide only imaging-derived, putative clearance-related markers. Current MRI measures do not directly measure tau or α-synuclein removal, and protein biomarker changes can arise from production, release, phosphorylation, redistribution, peripheral kinetics, or assay behavior. These measures should therefore not be overinterpreted as evidence that exercise removes misfolded proteins from the human brain.

Future experiments should therefore be designed as mechanistic perturbation studies. They should compare exercise timing under matched prescribed dose, distinguish completed workload from internal physiological load and adherence, measure 24-h movement behavior, sleep architecture, and circadian phase, evaluate vascular pulsatility and cerebrovascular function, use multimodal imaging-derived neurolymphatic surrogates, and include disease-specific protein biomarkers such as tau PET, plasma p-tau217, and α-synuclein seed amplification assays. The strongest target populations are those in which protein spread is detectable but not yet anatomically saturated, including amyloid-positive individuals with early tau pathology and people with prodromal synucleinopathy such as isolated REM sleep behavior disorder. A change in any single biomarker should not be interpreted as proof of clearance or mediation.

## Supplementary material

Supplementary Figure 1 Directed acyclic graph of the proposed exercise-timing pathway, distinguishing baseline confounders and effect modifiers, assigned timing exposure, post-assignment sleep/circadian/vascular mediators, implementation variables and co-exposures, imaging-derived neurolymphatic surrogates, disease-specific protein endpoints, and clinical outcomes.

Supplementary Table 1 Key human studies informing the proposed framework, including sample size, exposure and timing definition, principal effect estimate and uncertainty where reported, statistical adjustment or analysis strategy, and the main inferential limitation.
