# Supplementary material for: Exercise timing as a potential temporal regulator of neurolymphatic physiology in neurodegenerative proteinopathies: a mechanistic framework
Source: Front Neurosci. 2026 Sep 7;20:1879617. doi: 10.3389/fnins.2026.1879617 (PMC13591387; doi:10.3389/fnins.2026.1879617)
Supplement: Supplementary file 1 [file Supplementary_file_1.docx]

**Supplementary Material**

**Exercise timing as a potential temporal regulator of neurolymphatic physiology in neurodegenerative proteinopathies: a mechanistic framework**

**Chaowen Hou¹* • Yuxin Gao² • Hongyu Wang³**

¹ Qilu Institute of Technology, Jinan, China | ² Shandong University of Engineering and Vocational Technology, Jinan, China | ³ Guangxi Normal University, Guilin, China

*Corresponding author: Chaowen Hou. Author emails: Yuxin Gao, jiusideyouxiang@163.com; Hongyu Wang, why158908@163.com

**Supplementary Figure 1**

**Supplementary Figure 1 |** Directed acyclic graph of the proposed exercise-timing pathway, distinguishing baseline confounders and effect modifiers, assigned timing exposure, post-assignment sleep/circadian/vascular mediators, implementation variables and co-exposures, imaging-derived neurolymphatic surrogates, disease-specific protein endpoints, and clinical outcomes. Solid arrows indicate prespecified causal paths to test; dashed arrows indicate hypothesized or alternative paths. Temporal order alone does not establish mediation, and imaging measures remain surrogate endpoints.

**Supplementary Table 1**

Study-level evidence map for key human studies informing the mechanistic framework. The table records sample size, timing definition, principal effect estimate and uncertainty when reported, analysis strategy, and the main inferential limitation. It is a qualitative evidence map and is not intended for quantitative pooling.

| **No.** | **Domain** | **Study** | **Population / design** | **Sample size** | **Exposure / timing definition** | **Principal effect estimate and uncertainty** | **Adjustment / analysis strategy** | **Main inferential limitation** | **DOI / source URL** |
| --- | --- | --- | --- | --- | --- | --- | --- | --- | --- |
| **1** | Physical activity and AD protein dynamics | Yau et al. (2025) | Harvard Aging Brain Study; longitudinal observational cohort of cognitively unimpaired older adults | n=296 cognition; n=241 amyloid PET; n=172 tau PET | Baseline pedometer-derived mean daily steps; no exercise-session timing or circadian phase definition | With elevated amyloid, higher activity was associated with slower inferior-temporal tau accumulation (β=-0.13, 95% CI -0.19 to -0.06; p<0.001), slower PACC5 decline (β=0.10, 95% CI 0.05 to 0.16; p<0.001), and slower CDR-SOB progression (β=-0.14, 95% CI -0.22 to -0.05; p=0.001). No association with amyloid accumulation (β=-0.0006, 95% CI -0.01 to 0.01; p=0.92). | Linear mixed-effects models; age, sex, education and timing of tau PET included; sensitivity analyses addressed season, self-reported activity, vascular risk, depression, baseline outcomes and early MCI/dropout. | Observational; activity measured at baseline only; reverse causation and residual confounding remain; no exercise-timing, sleep-clearance or direct neurolymphatic measure. | https://doi.org/10.1038/s41591-025-03955-6 |
| **2** | Exercise and neurolymphatic imaging | Yoo et al. (2025) | Healthy volunteers; randomized single-bout and 12-week exercise groups with pre/post MRI | n=37 analyzed (single bout n=21; 12-week training n=16) | Cycling at 50% VO2max for the acute condition; 30 min, 3 times/week for 12 weeks, progressing from 45% to 60% VO2max; clock-time/circadian phase not specified | After 12 weeks, putamen ΔT1_influx increased from median 25.7 [24.3–34.2] to 34.7 [31.4–40.7] ms (p=0.01); basal-bone meningeal lymphatic ROI area increased from 15.0 [12.0–18.5] to 21.5 [19.0–26.9] mm² (p=0.008); mLV flow increased from 31.4 [22.3–42.4] to 36.6 [29.2–47.9] mm³/s (p=0.002). Acute single-bout changes were not significant. | Within-group two-sided Wilcoxon signed-rank tests; baseline group comparisons used Fisher exact/Mann–Whitney tests; proteomics used false-discovery-rate control. | Small healthy sample, single exercise modality, imaging surrogates rather than direct clearance, and no disease-specific protein or clinical endpoint. | https://doi.org/10.1038/s41467-025-58726-1 |
| **3** | Sedentary breaks and cerebral hemodynamics | Carter et al. (2018) | Healthy desk workers; randomized crossover laboratory study | n=15 (10 male) | Three 4-h conditions: uninterrupted sitting; 2-min light walking every 30 min; or 8-min light walking every 2 h | Change in middle cerebral artery velocity was -3.2±1.2 cm/s during uninterrupted sitting versus 0.6±1.5 cm/s with 2-min walks every 30 min (p=0.02); the 8-min/every-2-h condition was -1.2±1.0 cm/s (p=0.14 vs sitting). | Randomized crossover; repeated within-participant comparisons of cerebral blood-flow velocity, dynamic cerebral autoregulation and CO2 reactivity. | Acute, small, healthy sample; transcranial Doppler measures velocity rather than volumetric flow; no sleep, protein or neurodegenerative endpoint. | https://doi.org/10.1152/japplphysiol.00310.2018 |
| **4** | Morning exercise, sitting and cerebral hemodynamics | Wheeler et al. (2019) | Overweight/obese older adults; randomized three-condition crossover study | n=12 (2 female; mean age 70±7 y) | 8-h laboratory conditions: uninterrupted sitting; 30-min moderate morning walking after 1 h sitting; or the same morning exercise plus 3-min light walking every 30 min | Middle cerebral artery velocity declined linearly during sitting (p<0.001); the 8-h average was higher with morning exercise alone than with sitting (p=0.007) or exercise plus breaks (p=0.024). | Randomized crossover with bilateral transcranial Doppler assessed at 13 time points; temporal trend and average velocity compared across conditions. | Very small acute study; no sleep/circadian phase, disease biomarker or direct neurolymphatic outcome; Doppler velocity is a surrogate. | https://doi.org/10.1152/japplphysiol.00001.2019 |
| **5** | Sedentary breaks in older adults | Maasakkers et al. (2020) | Older adults; four-condition crossover study crossing sitting breaks with low/high mental activity | n=22 (mean age 78 y; 9 female) | 3 h sitting, with or without 2-min walking breaks every 30 min; low versus high mental activity | No condition differences in cognition, cerebral blood-flow velocity, baroreflex sensitivity, autoregulation or vasomotor reactivity. Blood pressure rose 8.6 mmHg (95% CI 5.0–12.1; p<0.001) and cerebrovascular resistance rose 0.23 (95% CI 0.01–0.45; p=0.04) over time, without mitigation by breaks. | Within-participant crossover comparisons; transcranial Doppler, Finapres and cognitive testing before/after each condition. | Acute exposure and small sample; null break effect in this protocol; no disease-specific or clearance endpoint. | https://doi.org/10.1186/s13195-020-00644-z |
| **6** | Sedentary breaks and cerebrovascular function | Tallon et al. (2023) | Healthy children aged 7–13 y; acute crossover sitting study | n=31 completed; analyzable kinetic data n=13 for MCA and n=8 for PCA/ICA | 3 h uninterrupted sitting versus sitting interrupted by hourly 10-min exercise breaks | Uninterrupted sitting reduced internal-carotid CO2 reactivity and slowed the middle-cerebral-artery velocity onset response; intermittent exercise breaks prevented the impairment. Exact primary contrast CI was not reported in the accessible summary. | Crossover comparisons with maturation included as a covariate; neurovascular coupling and cerebrovascular reactivity assessed using flow/velocity kinetics. | Pediatric, mostly prepubertal sample; substantial signal loss; no adult neurodegeneration, sleep or protein endpoint. | https://doi.org/10.1113/EP091314 |
| **7** | Evening exercise and sleep/autonomic recovery | Leota et al. (2025) | Large multinational free-living wearable cohort; observational person-night analysis | 14,689 physically active adults; 4,084,354 person-nights | Exercise end time relative to habitual/actual sleep onset, jointly modeled with exercise strain | Later and higher-strain exercise was dose-dependently associated with later sleep onset, shorter sleep, poorer sleep quality, higher nocturnal resting heart rate and lower HRV. Exercise ending ≥4 h before sleep was not associated with sleep changes. Light versus maximal strain ending 2 h before habitual sleep was associated with 36.0 min earlier sleep onset, 5.4% longer sleep, 6.8% lower nocturnal RHR and 14.1% higher HRV. | Generalized additive mixed models across repeated person-nights; exploratory effect modification by sex, age and BMI. | Observational wearable data in active adults; residual confounding by light, meals and behavior; no neurodegenerative or protein endpoint. | https://doi.org/10.1038/s41467-025-58271-x |
| **8** | Rest–activity rhythm and incident dementia/MCI | Haghayegh et al. (2024) | UK Biobank prospective cohort with wrist actigraphy and linked clinical records | n=91,517; 555 incident dementia/MCI events; follow-up up to 7.5 y | Six days of wrist actigraphy; nonparametric RAR measures (M10, L5, relative amplitude, stability, fragmentation) plus cosinor amplitude/acrophase | Per 1-SD decrease in relative amplitude, HR 1.23 (95% CI 1.16–1.29); per 1-SD decrease in M10, HR 1.28 (1.14–1.44); per 1-SD increase in intradaily variability, HR 1.14 (1.05–1.24); per 1-SD decrease in 24-h amplitude, HR 1.32 (1.17–1.49). | Cox models adjusted for age, sex, education, deprivation, ethnicity, obesity, sleep apnea, morbidity, circulatory disease, shift work, alcohol, smoking and AD polygenic risk; sensitivity and interaction analyses. | Single actigraphy window; diagnoses from records; >95% White European sample; intrinsic circadian phase and environmental light were not measured; reverse causation remains possible. | https://doi.org/10.2196/55211 |
| **9** | 24-h activity patterns and incident AD/PD | Winer et al. (2024) | UK Biobank community cohort with wrist accelerometry and longitudinal diagnosis/cognition | n=82,829; 187 incident AD; 265 incident PD; mean follow-up 6.8±0.9 y | One week of wrist accelerometry summarized using cosinor, nonparametric and functional principal-component measures | AD: diurnal amplitude HR 0.79 (95% CI 0.65–0.96) and M10 HR 0.75 (0.61–0.94) per SD increase. PD: diurnal amplitude HR 0.28 (0.23–0.34), mesor HR 0.13 (0.10–0.16), L5 HR 0.24 (0.08–0.69) and M10 HR 0.20 (0.16–0.25). | Cox regression adjusted for age, sex, education, general health, BMI and deprivation; matched functional principal-component analyses; longitudinal cognitive models. | Observational; diagnostic misclassification/under-ascertainment possible; no concurrent sleep log or circadian phase; activity impairment may be prodromal disease rather than causal. | https://doi.org/10.1186/s13195-024-01411-0 |
| **10** | Exercise and cognition/AD biomarkers | Baker et al. (2025; EXERT) | Phase 3 multicenter randomized controlled trial in sedentary older adults with amnestic MCI | Approximately 300 randomized; exact analysis-set size varies by endpoint | 18 months of moderate–high-intensity aerobic training versus low-intensity stretching/balance/range-of-motion exercise; supervised for 12 months; session clock-time not assigned | The trial established feasibility of long-duration structured exercise and assessed cognition and AD biomarkers. No exercise-timing contrast, sleep-gated clearance endpoint, or propagation estimand was prespecified. | Randomized group comparison with longitudinal cognitive and biomarker outcomes; endpoint-specific estimates should be taken from the published EXERT report. | Tests exercise modality/intensity, not time of day; active control may reduce between-group contrast; does not directly measure tau propagation or neurolymphatic clearance. | https://doi.org/10.1002/alz.14586 |
| **11** | High-intensity exercise feasibility in PD | Schenkman et al. (2018; SPARX) | Phase 2 multicenter randomized clinical trial in de novo Parkinson disease | n=128 (high intensity n=43; moderate n=45; wait-list n=40) | Treadmill exercise 4 days/week for 6 months at 80–85% or 60–65% maximum heart rate; no assigned clock-time | Six-month UPDRS motor change was 0.3 (95% CI -1.7 to 2.3) in the high-intensity group versus 3.2 (1.4 to 5.1) in usual care (p=0.03); high intensity met the prespecified nonfutility threshold. | Randomized three-arm phase-2 nonfutility design with masked clinical assessment; adherence, achieved intensity and safety were quantified. | Designed for feasibility/nonfutility, not definitive disease modification; no timing, sleep, α-synuclein or neurolymphatic endpoint. | https://doi.org/10.1001/jamaneurol.2017.3517 |
| **12** | Home aerobic exercise in PD | van der Kolk et al. (2019; Park-in-Shape) | Single-center, double-blind randomized controlled trial in mild Parkinson disease | n=130 randomized 1:1 | Home stationary-cycle aerobic exercise, 30–45 min, 3 times/week for 6 months, versus stretching/flexibility/relaxation; no assigned clock-time | Home-based, gaming-supported aerobic exercise improved motor outcomes and aerobic fitness relative to active control; exact primary effect and CI should be taken from the primary Lancet Neurology report. | Randomized active-control trial with remote supervision and masked assessment; intention-to-treat framework. | No time-of-day contrast and no sleep, α-synuclein or neurolymphatic endpoint; symptomatic and fitness effects cannot establish clearance or slowed propagation. | https://doi.org/10.1016/S1474-4422(19)30285-6 |
| **13** | Community aerobic exercise in PD | Uc et al. (2014) | Phase I/II randomized community-based aerobic exercise trial in Parkinson disease | n=60; 81% completed | Aerobic exercise program in a community setting; exercise clock-time/circadian phase not assigned | Mean attendance was 83.3% (95% CI 77.5–89.0) and mean achieved intensity was 46.8% heart-rate reserve (95% CI 44.0–49.7); no serious adverse events were reported. | Randomized early-phase trial emphasizing feasibility, fitness, motor, cognitive and quality-of-life outcomes. | Small early-phase study with attrition; no assigned timing, sleep, α-synuclein or clearance endpoint. | https://doi.org/10.1212/WNL.0000000000000644 |
| **14** | Exercise timing and blood pressure | Brito et al. (2019) | Randomized controlled aerobic-training study in treated hypertension | NR in the manuscript proof; verify against the primary report before final submission | Morning versus evening aerobic training under matched program conditions | Evening training produced a larger ambulatory blood-pressure reduction than morning training; exact endpoint-specific estimate and 95% CI should be transcribed from the primary report before production upload. | Randomized timing-group comparison with ambulatory blood-pressure and hemodynamic outcomes. | Cardiovascular population and surrogate physiology; no neurodegenerative, sleep-architecture, neurolymphatic or protein endpoint. | https://doi.org/10.1249/MSS.0000000000001852 |
| **15** | Exercise timing and autonomic control | Brito et al. (2024) | Controlled aerobic-training study in older adults with treated hypertension | NR in the manuscript proof; verify against the primary report before final submission | Morning versus evening aerobic training | Evening, but not morning, training improved sympathetic activity and baroreflex sensitivity; exact endpoint-specific estimate and 95% CI should be transcribed from the primary report before production upload. | Between-group training comparison of autonomic and baroreflex outcomes. | Indirect vascular/autonomic evidence; sample not selected for neurodegenerative disease; no direct sleep-clearance or protein outcome. | https://doi.org/10.1113/JP285966 |

**Notes.** NR, not reported in the manuscript proof or not reliably recoverable from the accessible primary-source summary; AD, Alzheimer's disease; CI, confidence interval; CSF, cerebrospinal fluid; DTI-ALPS, diffusion tensor image analysis along the perivascular space; HR, hazard ratio; HRV, heart-rate variability; ICA, internal carotid artery; M10/L5, most active 10 h / least active 5 h; MCA, middle cerebral artery; MCI, mild cognitive impairment; mLV, meningeal lymphatic vessel; PACC5, Preclinical Alzheimer Cognitive Composite; PD, Parkinson's disease; PET, positron emission tomography; PVS, perivascular space; RAR, rest–activity rhythm; SAA, seed amplification assay; UPDRS, Unified Parkinson's Disease Rating Scale.

**Editorial verification note.** Amber cells identify two Brito timing studies for which the proof did not provide a sample size or full endpoint-specific estimate. These entries are deliberately not inferred and should be checked against the authors' source PDFs before the journal's final upload.
